# Crystallographic Visualization of Distinct Iodic Aggregations in Isostructural Metal–Organic Frameworks

**DOI:** 10.1021/jacs.5c04910

**Published:** 2025-05-28

**Authors:** Yi Han, Yiwen He, Yin-Ke Fu, Hongliang Huang, Hongdong Li, Jiong-Peng Zhao, Lei Wang, Qian Niu, Nathaniel L. Rosi

**Affiliations:** † Key Laboratory of Eco-Chemical Engineering, Ministry of Education, International Science and Technology Cooperation Base of Eco-chemical Engineering and Green Manufacturing, 66280 College of Chemistry and Molecular Engineering Qingdao University of Science and Technology, Qingdao 266042, P. R. China; ‡ Department of Chemistry, University of Pittsburgh, Pittsburgh, Pennsylvania 15260, United States; § State Key Laboratory of Advanced Separation Membrane Materials, School of Chemical Engineering and Technology, 47847 Tiangong University, Tianjin 300387, P. R. China; ∥ School of Chemistry and Chemical Engineering, Tianjin Key Laboratory of Organic Solar Cells and Photochemical Conversion, 66346 Tianjin University of Technology, Tianjin 300384, P. R. China; ⊥ Department of Laboratory Medicine/Clinical Laboratory Medicine Research Center, West China Hospital, 12530 Sichuan University, Chengdu 610017, P. R. China; # Sichuan Clinical Research Center for Laboratory Medicine, Chengdu 610041, P. R China; ¶ Department of Chemical & Petroleum Engineering, University of Pittsburgh, Pittsburgh, Pennsylvania 15260, United States

## Abstract

Precisely determining the location of adsorbed molecules is essential for illuminating the mechanisms underlying molecular confinement within porous metal–organic frameworks (MOFs). Here, we present the pore-filling and reactive adsorption of iodine in ALP-MOF-1 and its isostructural redox-active ALP-MOF-2. The adsorbed iodine molecules (I_2_) are unaffected by Zn­(II) in ALP-MOF-1 and are exclusively confined into an unusual three-dimensional (3D) iodine aggregation due to the 3D cross-linked pore topology and multiple I_2_-framework interactions. Conversely, in ALP-MOF-2, the adsorbed I_2_ enables the oxidation of Co­(II) to Co­(III), which is accompanied by the reduction of I_2_ to I_3_^–^ and the formation of I_5_^–^ and I_2_ during continuous I_2_ loading. Identification of distinct iodine adsorption processes in ALP-MOF-1 and −2 motivated tuning of the metal ion composition to adjust the adsorption mechanism. The iodic aggregations in both MOFs are unambiguously confirmed by the combination of single crystal X-ray diffraction and spectroscopic characterization. The presence of multiple adsorption sites facilitate rapid iodine uptake of ∼179 wt % in ALP-MOF-1 and ∼150 wt % in ALP-MOF-2 within ∼5 h, which could be advantageous for applications requiring rapid and energy-efficient iodine capture.

## Graphical abstract

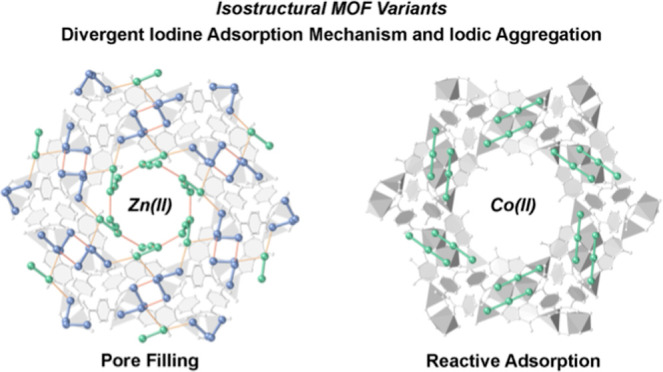

## Introduction

The confinement of guest molecules within porous metal–organic frameworks (MOFs)^1,2^ has been extensively investigated.^3–6^ Single crystal X-ray diffraction (SCXRD) analysis allows for precise determination of the location of included guests^7–15^ which is essential for understanding adsorption mechanisms at the atomic level. Additionally, redox-active metal centers and pore geometries can dramatically contribute to the absorptive behavior and potentially alter the spatial arrangement of included guests.^16,17^

^129^I and ^131^I are radioactive gaseous fission products that pose significant risks to human health and the environment,^18,19^ underscoring the need for methods and materials that enable their efficient capture. In this context, MOFs, with their high surface areas and tailorable pore environments, have been extensively explored and some exhibit superior adsorption capacities.^17,20–31^ Here, we present the crystallographic visualization of pore-filling and reactive adsorption of iodine (I_2_) in Zn-based ALP-MOF-1 and isostructural, redox-active Co-based ALP-MOF-2. In ALP-MOF-1, the adsorbed iodine molecules are unaffected by Zn­(II) and are exclusively confined by the 3D cross-linked pore geometry into a unique and unprecedented 3D aggregated iodine network, when compared to the 1D triple-helical iodine chains within MFM-300­(Sc)^17^ and well-known 2D iodine solid structure.^32^ In ALP-MOF-2, oxidation of Co­(II) leads to reduction of the adsorbed I_2_ to I_3_^–^ and further formation of I_5_^–^ and I_2_ during continuous I_2_ loading. Through experimental observations and computational studies, we identify the mechanism by which metal ions and metal ion composition affect iodine adsorption in ALP-MOFs, which is relevant to forming and controlling the ratios of distinct iodic aggregations. The high iodine uptakes of ∼ 179 wt % in ALP-MOF-1 and ∼ 150 wt % in ALP-MOF-2 within ∼5 h point toward potential applications in rapid and energy-efficient capture of radioactive iodine.

## Results and Discussion

### MOF Synthesis and Iodine Adsorption Studies

ALP-MOF-1 and ALP-MOF-2, along with their fully evacuated and activated states were prepared using our established protocols.^33^ ALP-MOF-1 consists of interconnected Zn_4_(μ_2_-H_2_O)_2_(ALP)_2_(BDC)_2_ clusters; the isostructural Co­(II) framework analogue, ALP-MOF-2, is obtained via metal ion metathesis without any noticeable porosity loss.^33^ Both MOFs adopt 3D ecz networks and exhibit 3D cross-linked pore systems with two pores having diameters of approximately 9.5 and 12 Å (Figures 1A and S1). In previous studies, we have demonstrated through sorption, spectroscopy, and crystallography that the relative Lewis acidities of metal sites in these MOFs dramatically affects water adsorption properties.^33^ Accordingly, we reasoned that ALP-MOF-1 and ALP-MOF-2 could be promising candidates for capture of radioactive iodine.

**Figure 1:**
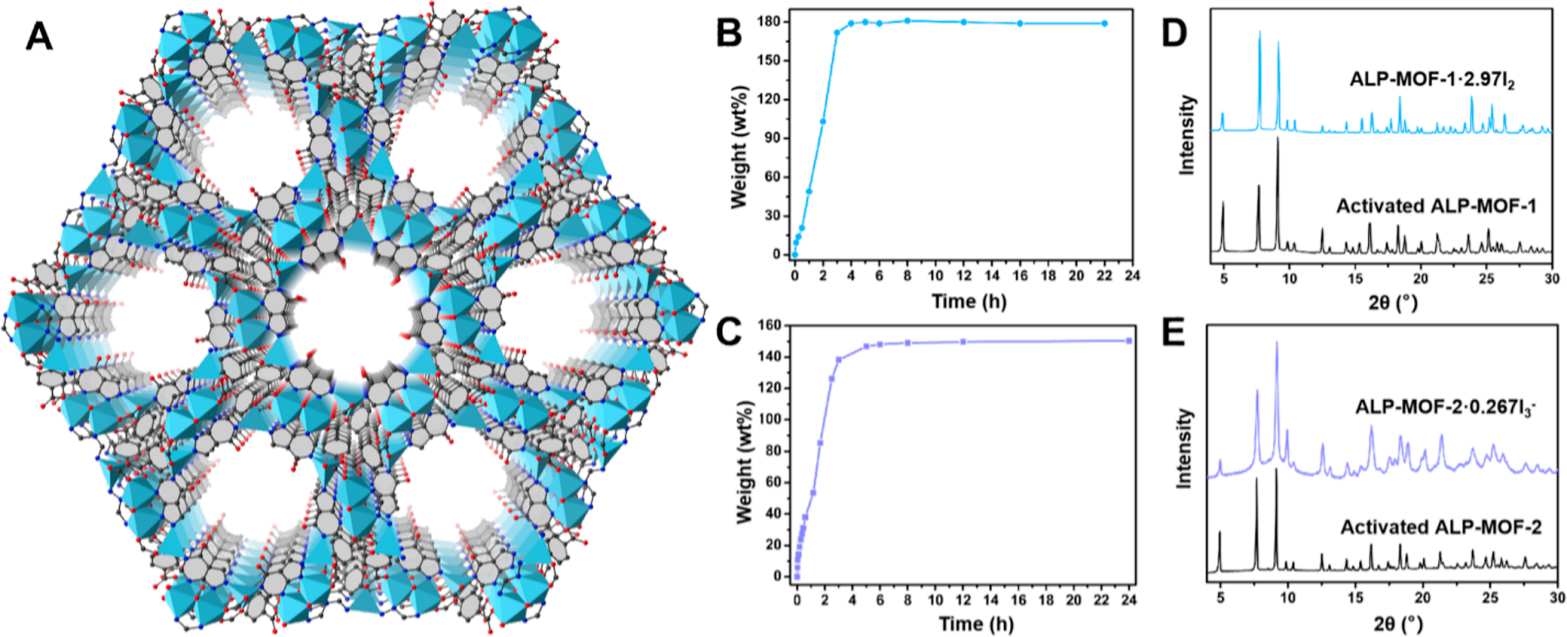
Single crystal structure, iodine adsorption isotherms, and PXRD patterns. (A) 3D crystal structure of ALP-MOF-1 and −2 viewed along the *c* crystallographic axis (Zn­(II) or Co­(II), blue octahedra; O, red sphere; N, blue sphere; C, black sphere; H atoms and guest solvent molecules are omitted for clarity). (B,C) Gravimetric iodine adsorption in ALP-MOF-1 and −2 as a function of time. (D,E) PXRD patterns for activated ALP-MOF-1/2 and ALP-MOF-1·2.97I_2_ and ALP-MOF-2·0.267I_3_^–^.

Adsorption experiments were conducted through diffusion of sublimated iodine vapor into activated ALP-MOF-1 and ALP-MOF-2, resulting in visual darkening of the crystalline materials (Figure S2). Scanning electron microscopy (SEM) confirmed the absence of adsorbed iodine on the crystal surfaces, and energy-dispersive X-ray spectroscopy (EDS) mapping revealed a uniform distribution of iodine throughout the MOF crystals (Figures S3 and S4). A quantitative study of iodine adsorption was performed gravimetrically by measuring the masses of iodine-loaded samples over time. For ALP-MOF-1, a steep rise in iodine uptake was observed between 0 and 3 h and apparent saturation was reached after ∼4 h, resulting in a maximum iodine uptake of ∼179 wt % (Figure 1B). A similar steep uptake was observed for ALP-MOF-2, while adsorption saturation and a maximum adsorption capacity of ∼150 wt % was achieved after ∼5 h (Figure 1C). These iodine adsorption capacities are higher than, or comparable to, reported MOFs including MFM-300­(Sc) (154 wt %),^17^ ZIF-8 (125 wt %),^34^ HKUST-1 (175 wt %),^35^ and [(ZnI_2_)_3_(TPT)_2_]·5.5­(C_6_H_5_NO_2_) [TPT = 2,4,6-tris­(4-pyridyl)-1,3,5-triazine] (175 wt %).^22^ The steep increase in uptake within the first 5 h supports the potential use of ALP-MOF-1 and ALP-MOF-2 for rapid iodine capture applications.

To unambiguously identify the molecular MOF···I and I···I interactions, we exposed activated crystals of ALP-MOF-1 to iodine vapor for 12 h and conducted single crystal X-ray diffraction (SCXRD) studies on the I_2_-loaded sample. Large peaks of electron density appeared within the pores, corresponding to four crystallographically unique iodine molecules with occupancies of approximately 61% (I_1_–I_2_), 67% (I_3_–I_4_), 82% (I_5_–I_6_), and 86% (I_7_–I_8_) (Figure 2A). Accordingly, this material is designated as ALP-MOF-1·2.97I_2_, formulated as [Zn_2_(ALP)­(BDC)]·2.97I_2_. The presence of 2.97 I_2_ molecules, equivalent to 176 wt %, matches well with the maximum iodine uptake and thermogravimetric analysis (TGA) results (Figure S5a). Powder X-ray diffraction (PXRD) confirmed that ALP-MOF-1·2.97I_2_ maintains its crystallinity (Figure 1D). The characteristic Raman band at 180 cm^–1^, assigned to iodine, corresponds to the I–I vibration frequency and is comparable to that of solid iodine (Figure S6a). X-ray photoelectron spectroscopy (XPS) indicated peaks at ∼619.8 and ∼631.1 eV, corresponding to I 3d_5/2_ and I 3d_3/2_, respectively (Figure S6b).

**Figure 2:**
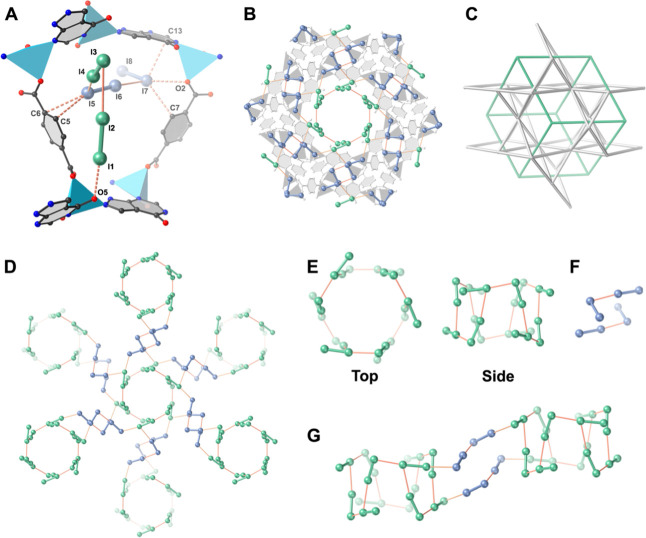
Crystallographic visualization of adsorbed iodine molecules and the 3D iodic aggregation confined in ALP-MOF-1 (Brown dashed and solid lines represent MOF···I and I···I interactions, respectively). (A) ALP-MOF-1·2.97I_2_ and the associated MOF···I and I···I interactions formed after exposure to iodine vapor for 12 h (2.97 iodine molecules per asymmetric unit). (B) Extended iodine network consisting of interconnected iodine molecules (green and blue). (C) Topological observation of 3D pcu iodine network within 3D ecz ALP-MOF-1 (green and gray skeletons represent iodine and MOF network, respectively.). (D) Extended iodine network alone, without MOF. (E) Top and side views of the cylinder-shaped I24 cluster formed from alternating I_1_–I_2_ and I_3_–I_4_ linked by I_1_···I_3_ and I_2_···I_3_ interactions. (F) The L-shaped I4 bridge formed from I_5_–I_6_ and I_7_–I_8_ linked by I_6_···I_7_ interactions. (G) Neighboring I24 clusters are connected through two I4 bridges.

The first iodine molecule, denoted I_1_–I_2_, is located close to the carbonyl oxygen of ALP, which serves as an ″anchoring″ site for a strong halogen bond (XB) between the framework and iodine (O5···I1 = 2.854 Å, ∠I2–I1–O5 = 174.7°).^36^ Fourier-transform infrared (FTIR) spectroscopy reveals a significant dampening of the ALP carbonyl stretch at ∼1700 cm^–1^ in ALP-MOF-1·2.97I_2_, confirming the strong interaction to adsorbed I_1_–I_2_ (Figure S7). The second iodine molecule, I_3_–I_4_, is positioned through intermolecular interaction with I_1_–I_2_ (I_2_···I_3_ = 3.805 Å). I_5_–I_6_, is situated between a phenyl ring (I_5_···C_5_ = 3.567 Å, I_5_···C_6_ = 3.497 Å) and I_3_–I_4_ (I_5_···I_4_ = 3.556 Å). Finally, I_7_–I_8_, interacts with I_5_–I_6_ (I_7_···I_6_ = 3.437 Å), a carboxyl O (I_7_···O_2_ = 3.335 Å), a phenyl ring (I_7_···C_7_ = 3.664 Å), and the pyrimidine ring of ALP (I_7_···C_13_ = 3.647 Å). The multiple Lewis basic adsorption sites in ALP-MOF-1, which combine oxygen-containing organic moieties and electron-rich π-systems synergistically facilitate I_2_ adsorption and immobilization and allow for their crystallographic visualization (Figures 2 and S8).

The 3D cross-linked pore system of ALP-MOF-1 contributes to the aggregation of confined iodine molecules into an unusual 3D iodine network with pcu topology (Figure 2B,C). This network contains 6-connected cylinder-shaped I24 clusters (Figure 2D,E), which are linked together through double L-shaped I4 bridges (Figure 2F,G) via strong interiodine molecular interactions (I_4_···I_5_, I_4_···I_8_ = 3.802 Å) (Figure S8f). Six alternating groups of I_1_–I_2_ and I_3_–I_4_ link together via I_1_···I_3_ (3.362 Å) and I_2_···I_3_ interactions to form the I24 cluster with *S*_6_ point group symmetry, located in the larger pore along the *c* crystallographic direction (Figure S8b–d). Twelve I4 bridges, consisting of one I_5_–I_6_ and one I_7_–I_8_ connected through I_6_···I_7_ interaction, extend from the I24 cluster (Figure 2D) and sit in the smaller pores (Figure S8e). Notably, 3D anionic polyiodide networks, such as hexacosaiodides (I_26_^3–^) and nonacosaiodides (I_29_^3–^), have been previously observed using template synthesis combined with distinct organic counterions.^37,38^ However, the unambiguous observation of this complex 3D iodine network represents the first example of such a motif exclusively resulting from geometrical confinement within a porous material.

To gain insight into the configuration and formation of the 3D iodine network, we utilized density functional theory (DFT) calculations^39,40^ in combination with independent gradient model (IGM) analysis.^41^ Four crystallographically unique iodine molecules, I_1_–I_2_, I_3_–I_4_, I_5_–I_6_, and I_7_–I_8_, observed via SCXRD, were considered as four distinct adsorption sites A-D respectively (Figure 2A). DFT calculations revealed binding energies of −51.69, −39.10, −60.03, and −65.06 kJ/mol for these sites. The 3D iso-surfaces developed using the IGM methodology clearly indicate MOF···I interactions, as revealed by the circular blue patches for I···carbonyl O of the ALP moiety and large green patches for regular I···carboxyl O atom and I···aromatic ring interactions (Figure 3A). Moreover, IGM data provided evidence for relatively strong intramolecular I···I interactions (Figure 3B and Section 2.2 in Supporting Information). The integrated MOF···I and I···I interactions thus allow for the stabilization of the complex 3D iodine network within ALP-MOF-1.

**Figure 3:**
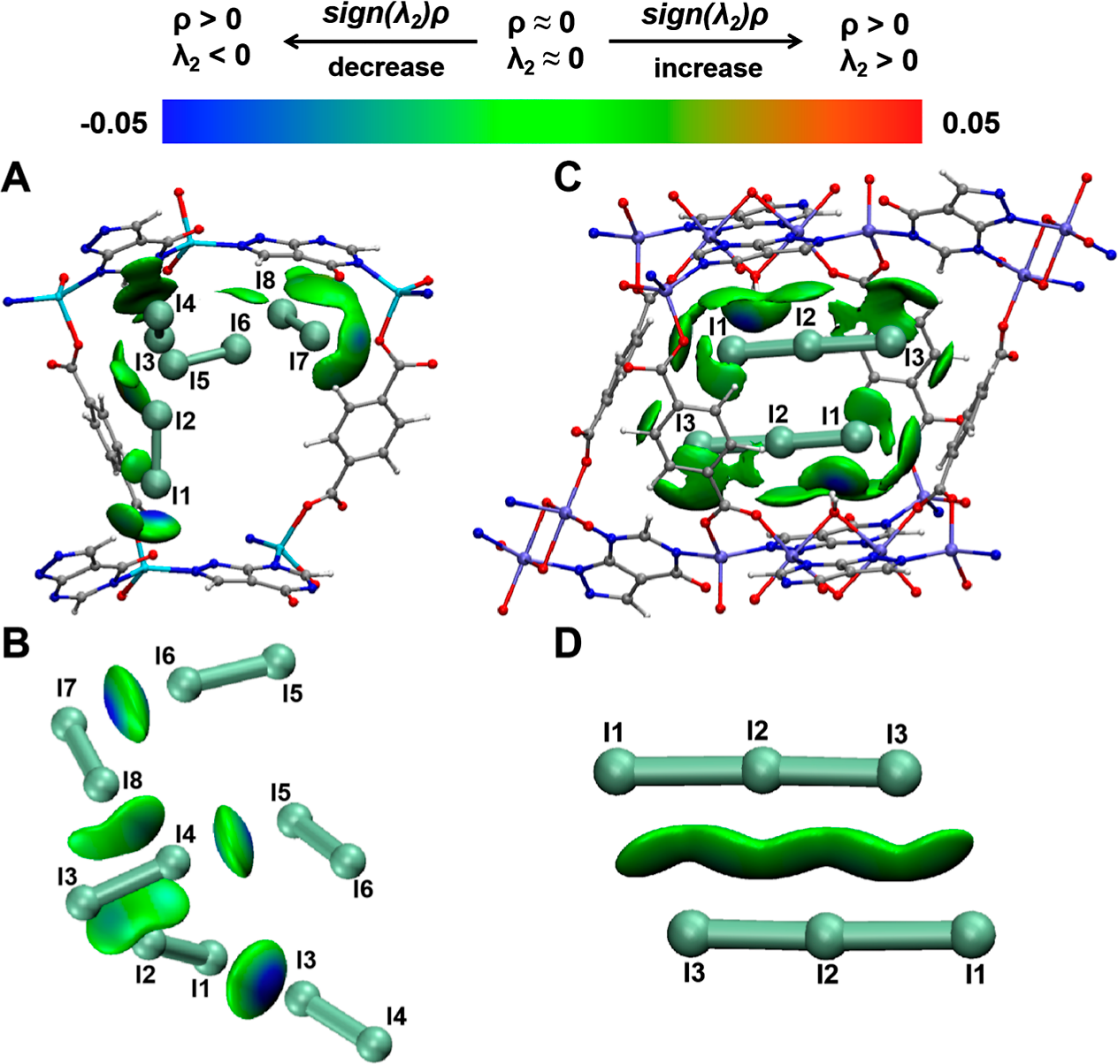
IGM analysis for the MOF···I and I···I interactions. (A,B) 3D iso-surfaces colored by sign of (λ_2_)­ρ for ALP-MOF-1·2.97I_2_ and (C,D) ALP-MOF-2·0.267I_3_^–^. (ρ is electron density, and λ is the second derivative (Laplacian) of the density ∇2ρ. Blue iso-surfaces indicate a strong attraction, green iso-surfaces indicate typical interactions, and red iso-surfaces indicate strong repulsion.).

Co­(II) in the isostructural ALP-MOF-2 significantly impacts water adsorption properties, due to its stronger affinity for water compared to Zn­(II).^33^ A recent SCXRD study revealed that open Co­(II) sites in Co_2_(*p*-DOBDC) can serve as coordinating sites for unique iodine chemisorption.^24^ Therefore, we anticipated that Co­(II) would coordinate strongly with iodine molecules, significantly affecting the iodine adsorption behavior. We exposed activated ALP-MOF-2 crystals to iodine vapor and used SCXRD to identify the adsorbed iodine. After 2 h of exposure, ALP-MOF-2 crystals were still crystalline yet weakly diffracting, as evidenced by PXRD; however, after 5 h of exposure and approaching iodine saturation, the crystallinity was almost completely lost (Figure S9b). A shorter exposure time of 20 min yielded acceptable SCXRD data (Figure 4A,B). Three large peaks of electron density with a closely linear arrangement (178.21°) appeared in the smaller pores, which we assigned to typical triiodides (I_3_^–^: I_1_–I_2_–I_3_) with an overall occupancy of ∼26.7%. Additionally, a bridging water molecule linked the two central Co ions together, because activated ALP-MOF-2 readily adsorbs water from the air at low relatively humidity.^33^ Thus, I_3_^–^-loaded ALP-MOF-2 is denoted as ALP-MOF-2·0.267I_3_^–^. The average bond length of I–I in I_3_^–^ is 2.91 Å, which is close to the value of 2.92 Å reported in the Cambridge Structural Database.^42^

**Figure 4:**
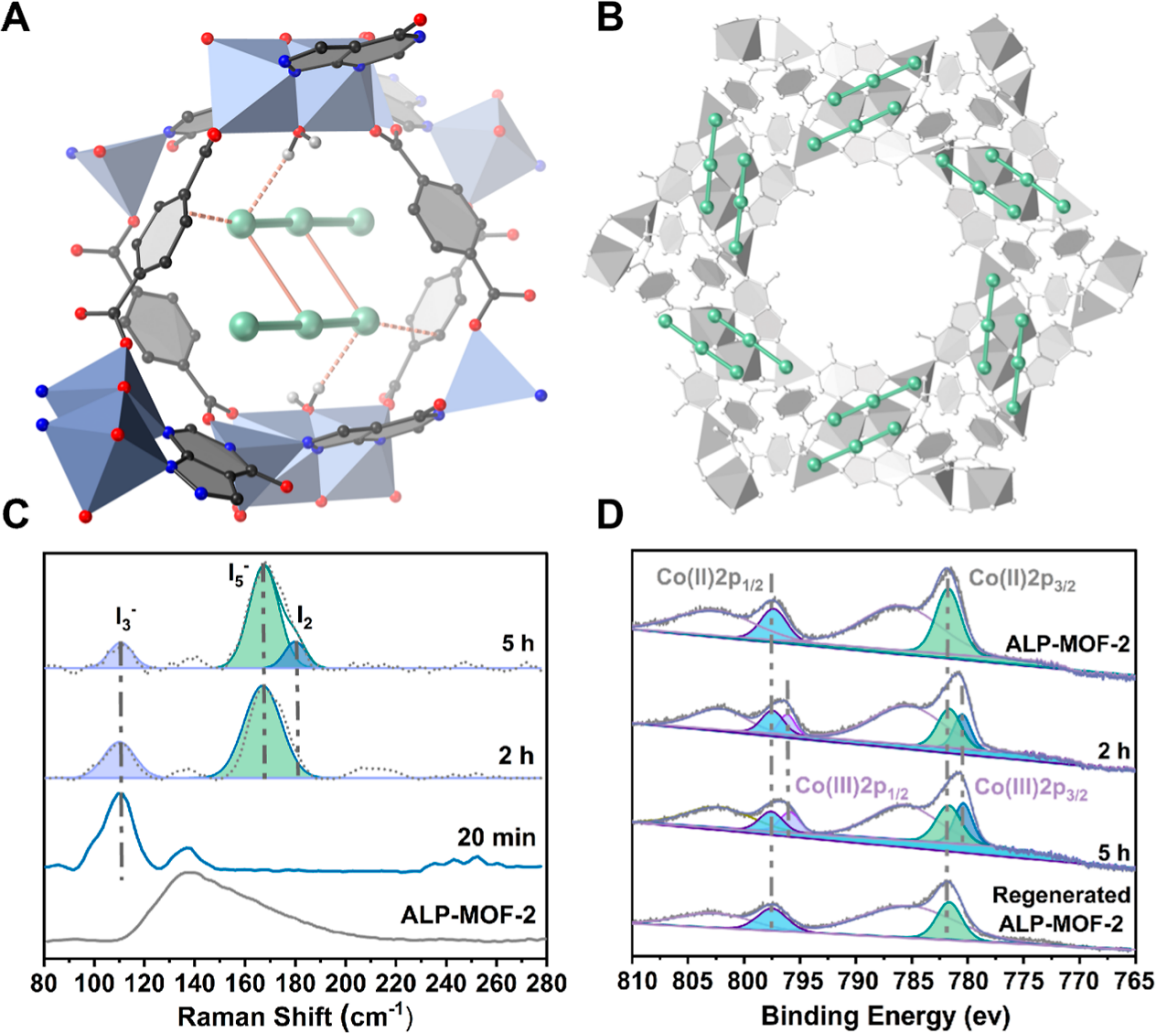
Crystallographic visualization of triiodides I_3_^–^ confined in ALP-MOF-2 and spectroscopic characterization of iodic species and cobalt. (A) ALP-MOF-2·0.267I_3_^–^ and the polyiodide (I_3_^–^)_2_ grid locating in the smaller pore formed after exposure to iodine vapor for 20 min (0.267I_3_^–^ per asymmetric unit, golden dashed and solid lines represent MOF···I and I···I interactions, respectively.). (B) Polyiodide (I_3_^–^)_2_ grids form along the framework backbone, leading to the larger pores totally empty. (C) Comparison of Raman spectra of ALP-MOF-2 after exposure to iodine vapor for 20 min (ALP-MOF-2·0.267I_3_^–^), 2 h (ALP-MOF-2·0.2I_3_^–^·0.6I_5_^–^), and 5 h (ALP-MOF-2·0.167I_3_^–^·0.833I_5_^–^·0.167I_2_). (D) The Co 2p XPS of ALP-MOF-2 after exposure to iodine vapor for 2 and 5 h, and regenerated ALP-MOF-2. Full spectra are shown in Figure S10.

The triiodides interact with the framework through Lewis acid–base interactions with the phenyl ring (I_1_···C_4_ = 3.529 Å) and hydrogen bonding with the bridging water molecule (HOH···I = 2.695 Å) (Figure 4A). Typically, triiodides form T-shaped^43^ or zigzag patterns^44^ in stacked structures or structures with channels. However, in ALP-MOF-2, dual I_1_···I_2_ (3.856 Å) interactions in neighboring I_3_^–^ units assist in linking two triiodides to form an unprecedented polyiodide (I_3_^–^)_2_ grid located in the smaller pores, leaving the larger pores empty (Figures 4B and S11). IGM analysis showed circular blue patches, indicating strong hydrogen bonding between triiodides and bridging water molecules (Figure 3C). Intramolecular I···I interactions observed within the polyiodide (I_3_^–^)_2_ grid are relatively weak type I interactions when compared to the unambiguous identifications of typical XBs in ALP-MOF-1·2.97I_2_ (Section 2.2 in Supporting Information). The binding energy of each triiodide in the entire MOF unit cell is −274.2 kJ/mol. PXRD reveals that ALP-MOF-2·0.267I_3_^–^ remains highly crystalline (Figure 1E), and TGA data closely aligns with the theoretical iodine loading value determined by SCXRD (Figure S5b). The Raman spectra, more reliable than XPS for distinguishing polyiodide species,^20^ show the characteristic band at ∼110 cm^–1^, consistent with the symmetrical stretch of I_3_^–^ in ALP-MOF-2·0.267I_3_^–^ (Figure 4C).

Co 2p XPS was used to understand the mechanism of I_3_^–^ formation within ALP-MOF-2. After 2 h of iodine vapor exposure, the spectrum shows an intense Co 2p_3/2_ photoelectron peak with shakeup satellite peaks. These can be deconvoluted into two contributions at ∼781.8 and 780.5 eV, corresponding to Co­(II) 2p_3/2_ and Co­(III) 2p_3/2_, respectively. For activated ALP-MOF-2, characteristic peaks for Co­(III) are absent (Figure 4D). We can therefore conclude that iodine adsorption in ALP-MOF-2 involves formation of polyiodide due to partial oxidation of Co­(II) to Co­(III) by adsorbed iodine, excluding the possibility of charge transfer between the phenyl ring and polyiodide.^45^ Accordingly, ALP-MOF-2·0.267I_3_^–^ (20 min iodine exposure time) determined by SCXRD can be more precisely formulated as [Co^III^_0.267_Co^II^_1.733_(μ_2_-H_2_O)­(ALP)­(BDC)]·0.267I_3_^–^. For the sample exposed to iodine for 2 h, the integration of the Co­(III) 2p_3/2_ and Co­(II) 2p_3/2_ peaks gives a Co­(III)/Co­(II) ratio of ∼1/1.5, potentially equating to the formula of [Co^III^_0.8_Co^II^_1.2_(μ_2_-H_2_O)­(ALP)­(BDC)]·0.8I_3_^–^ (ALP-MOF-2·0.8I_3_^–^, 2 h). This corresponds to an iodine adsorption capacity of ∼ 70.2 wt %, which is significantly lower than the experimental gravimetric value (∼102 wt %). The Raman spectrum collected after 2 h of iodine exposure revealed a large new band at ∼167 cm^–1^ appearing near the characteristic I_3_^–^ band, which can be attributed to the vibrations of I_5_^–^ (Figure 4C).^45^ Careful integration of these peaks yielded I_3_^–^/I_5_^–^ ratio of ∼1/3 (Figure S12a). Based on this analysis, the composition of ALP-MOF-2 after 2 h exposure to iodine vapor can thus be corrected to [Co^III^_0.8_Co^II^_1.2_(μ_2_-H_2_O)­(ALP)­(BDC)]·0.2I_3_^–^·0.6I_5_^–^ (ALP-MOF-2·0.2I_3_^–^·0.6I_5_^–^), corresponding to ∼105 wt % iodine, which is much closer to the experimental value. We next collected Raman and Co 2p XPS spectra for ALP-MOF-2 saturated with iodine (after 5 h of iodine exposure), even though this sample nearly lost crystallinity. The Raman spectrum revealed the characteristic I_3_^–^ band and a broad band within ∼155–185 cm^–1^ (Figure 4C), which can be divided into the I_5_^–^ and I_2_ characteristic bands at ∼180 cm^–1^. Peak integration yields a I_3_^–^/I_5_^–^/I_2_ ratio of ∼1/5/1 (Figure S12b). The Co 2p XPS spectrum showed the coexistence of Co^III^ and Co^II^ with a slightly higher ratio of ∼1/1. From these data, saturated ALP-MOF-2 is formulated as [Co^III^Co^II^(μ_2_-H_2_O)­(ALP)­(BDC)]·0.167I_3_^–^·0.833I_5_^–^·0.167I_2_ (ALP-MOF-2·0.167I_3_^–^·0.833I_5_^–^·0.167I_2_). This formula corresponds to an adsorption capacity of ∼146 wt % (5 h), consistent with the experimental gravimetric value of ∼150 wt %.

The reversibility of iodine adsorption was determined by soaking ALP-MOF-1·2.97I_2_ and ALP-MOF-2·0.167I_3_^–^·0.833I_5_^–^·0.167I_2_ in ethanol and characterizing the samples using time-dependent UV–vis spectra, EDS mapping data, and photographs (Figure S3c, S4c, and S13). EDS-mapping data showed negligible iodine content in both samples after soaking in ethanol, indicating that the included iodic species can be readily released. Importantly, the regenerated ALP-MOF-2, namely ALP-MOF-2·2H_2_O, in which Co­(III) fully reverted to Co­(II) (Figure 4D), recovered its crystallinity, and its structure was confirmed by PXRD and SCXRD (Figures S9b and S14). Both ALP-MOFs are recyclable with ethanol rinsing (ALP-MOF-1, 120 h; ALP-MOF-2, 180 h) and subsequent thermal activation. The iodine adsorption capacities of both samples were maintained after four cycles (Figure S15).

ALP-MOF-1 and -2 have similar iodine adsorption isotherms yet apparently different adsorption mechanisms: ALP-MOF-1 exhibits gradual iodine uptake via a pore-filling process while Co­(II) in ALP-MOF-2 results in reactive adsorption of iodine. In addition, I_2_ adsorbs more rapidly at early times (<0.3 h) for ALP-MOF-2 vs ALP-MOF-1 (Figure S20), which is supported by the overall binding energy of triiodide and iodine in entire MOF unit cell (−274.2 kJ/mol vs −215.8 kJ/mol). This could be related to the reductive loading process, which would create electrostatic interactions between MOF framework and I_3_^–^. These results and observations motivated adjustment of the Zn/Co ratio to investigate the adsorption mechanism transition from pore-filling to reactive loading. Isolation of crystalline analogues by incomplete metathesis of Zn­(II) with Co­(II) were achieved with the following Zn/Co ratios: 0.24:0.76, 0.58:0.42 and 0.89:0.11, as determined by SEM-EDS (Figure S21).^33^ Raman spectra after adsorption saturation indicate both the presence of I_3_^–^ and a decreasing amount of I_2_ with increasing formation of I_5_^–^ as the ratio of Zn/Co transitions from <1:0 to 0:1 (Figure S22a). These data demonstrate that rational adjustment of metal ion composition enables careful and deliberate tuning of iodic aggregations and their ratios. Interestingly, Raman spectra for the 0.89:0.11 analogue after 20 min of iodine exposure closely resembles ALP-MOF-2 (20 min) in terms of the presence of I_3_^–^ and absence of I_2_ signals, suggesting that the reactive adsorption mechanism is preferred in Co­(II)-included MOFs, even when Co­(II) is minimally incorporated (Figure S22b).

## Conclusions

Our experimental and computational investigations have revealed the pore-filling and reactive adsorption of large amounts of iodine in ALP-MOF-1 and its isostructurally redox-active ALP-MOF-2, respectively. The redox-active metal centers and multiple adsorption sites within both MOFs facilitate the crystallographic visualization of distinct iodic species. The 3D cross-linked pore system in ALP-MOF-1 leads to the formation of an unprecedented 3D complex iodine network through strong intramolecular halogen bonds (XBs). Conversely, in isostructural ALP-MOF-2, I_2_ are reduced by Co­(II) to I_3_^–^ and further loading generates I_5_^–^/I_2_ species, even when Co­(II) is only present in small amounts. Adjustment of metal ion composition enables dramatic tuning of the iodic aggregations and their ratios. Collectively, the results provide insight into chemical and structural features that affect iodine aggregation in confined environments and thus may influence the design of iodine capture materials. Finally, we emphasize that the recyclable absorptive capabilities of ALP-MOF-1 and -2 underscore their potential as efficient sorbents for the rapid and energy-efficient capture of radioactive iodine.

## Experimental Methods

### Synthesis of MOFs

ALP-MOF-1 and ALP-MOF-2, along with their fully activated states, were prepared using established protocols.^33^

### Iodine Uptake Experiments

#### ALP-MOF-1·2.97I_2_

A 4 mL uncapped vial containing single crystals of activated ALP-MOF-1 (∼15 mg) was rapidly transferred into a 20 mL vial containing solid iodine. The 20 mL vial was then capped and allowed to stand at 50 °C for 12 h. EA calcd (%) for Zn_2_(ALP)­(BDC)*·*2.97I_2_ (C_13_H_6_N_4_O_5_I_5.94_Zn_2_): C, 13.18; H, 0.51; N, 4.73; found: C, 12.94; H, 0.66; N, 4.53.

#### ALP-MOF-2·0.267I_3_^–^

A 4 mL uncapped vial containing single crystals of activated ALP-MOF-2 (∼15 mg) was rapidly transferred into a 20 mL vial containing solid iodine. The 20 mL vial was then capped and allowed to stand at 50 °C for either 20 min, 2 h, or 5 h. The molecular formula for the 20 min sample was determined: EA calcd (%) for [Co^III^_0.267_Co^II^_1.733_(μ_2_-H_2_O)­(ALP)­(BDC)]·0.267I_3_^–^ (C_13_H_8_N_4_O_6_I_0.8_Co_2_): C, 29.13; H, 1.49; N, 10.46; found: C, 30.81; H, 1.93; N, 9.87., ALP-MOF-2·0.2I_3_^–^·0.6I_5_^–^ (for 2 h); ALP-MOF-2·0.167I_3_^–^·0.833I_5_^–^·0.167I_2_ (for 5 h).

#### Regeneration Experiments

The ALP-MOF-1·2.97I_2_ sample was soaked in ethanol (4 mL) for 120 h. Each day during this process, the ethanol was removed and replaced with fresh ethanol (4 mL).

The ALP-MOF-2·0.167I_3_^–^·0.833I_5_^–^·0.167I_2_ sample was soaked in ethanol (4 mL) for 180 h. Each day during this process, the ethanol was removed and replaced with fresh ethanol (4 mL). Iodine content in the ethanol was determined using UV–vis spectroscopy.

#### Mixed Zn­(II)/Co­(II) ALP-MOFs

Incubating as-synthesized ALP-MOF-1 single crystals (∼30 mg) in a CH_3_CN solution of CoCl_2_ (20 mL, 0.01 M) at room temperature can afford analogues with the following Zn/Co ratios (note: incubation time in parentheses): 0.89:0.11 (∼0.5 h), 0.58:0.42 (∼12 h), 0.76:0.24 (∼24 h). The Zn/Co ratios were determined using SEM-EDS on thoroughly ground samples of the MOF crystals.

## Supplementary Material



## Notes

SEM-EDS; TGA; PXRD; SCXRD; Raman, XPS, UV–vis and FT-IR spectroscopy; and computational methods (PDF)
