# Supplementary material for: Crystallographic Visualization of Distinct Iodic Aggregations in Isostructural Metal–Organic Frameworks
Source: J Am Chem Soc. 2025 May 28;147(23):19968–75. doi: 10.1021/jacs.5c04910 (PMC12164258; doi:10.1021/jacs.5c04910)
Supplement: Supplementary file 1 [file ja5c04910_si_001.pdf]

Crystallographic Visualization of Distinct Iodic Aggregations in Isostructural Metal−Organic Frameworks

Yi Han[a]*, Yiwen He[b], Yin-Ke Fu[a], Hongliang Huang[c]*, Hongdong Li[a], Jiong-Peng Zhao[d], Lei Wang[a]*, Qian Niu[e], and Nathaniel L. Rosi[b,f]*

[a]*Key Laboratory of Eco-Chemical Engineering, Ministry of Education, International Science and Technology Cooperation Base of Eco-chemical Engineering and Green Manufacturing, College of Chemistry and Molecular Engineering Qingdao University of Science and Technology, Qingdao, P. R. China*
[b]*Department of Chemistry, University of Pittsburgh, Pittsburgh, Pennsylvania, United States*
[c]*State Key Laboratory of Advanced Separation Membrane Materials, School of Chemical Engineering and Technology, Tiangong University, Tianjin 300387, P. R. China*
[d]*School of Chemistry and Chemical Engineering, Tianjin Key Laboratory of Organic Solar Cells and Photochemical Conversion, Tianjin University of Technology, Tianjin, P. R. China*
[e]*Department of Laboratory Medicine/Clinical Laboratory Medicine Research Center, West China Hospital, Sichuan University, Chengdu, P. R. China, Sichuan Clinical Research Center for Laboratory Medicine, Chengdu, P. R China*
[f]*Department of Chemical & Petroleum Engineering, University of Pittsburgh, Pittsburgh, Pennsylvania, United States*

*E-mail: roberthan@yeah.net; huanghongliang@tiangong.edu.cn; inorchemwl@126.com; nrosi@pitt.edu

Table of Contents

Section	Page(s)
**1. Materials and Methods**	**2-4**
1.1 Materials	2
1.2 General Characterization Techniques	2-4
**2. Iodine Adsorption Studies**	**5-25**
2.1 Characterizations	5-21
2.2 Identification of I···I interactions	22-25
**3. Computational Methods**	**26-28**
**4. Metal Ion Composition Characterization**	**29-33**
**5. Crystallographic Data**	**34-37**
5.1 Asymmetric Unit ORTEP Diagrams	34
5.2 Crystallographic Tables	35-37

Section 1. Materials and Methods

1.1 Materials

$Zn(NO_3)_2 \cdot 6H_2O$ (98%), $CoCl_2 \cdot 6H_2O$ (98%), I_2 (≥ 99.8%), dimethylacetamide (DMA) (≥ 99%) and acetonitrile (CH_3CN) (≥ 99.5%) were purchased from Sigma-Aldrich. Allopurinol (H_2ALP) (> 98.0%) and terephthalic acid (H_2BDC) (> 99.0%) were purchased from TCI. Acetone (> 99.5%) and ethanol (> 95.0%) was purchased from Sinopharm Chemical Reagent Co., Ltd. NANOpure® (Thermo Scientific, > 18.2 MΩ · cm) water was used in the synthesis of the MOFs. All purchased chemicals were used without further purification.

1.2 General characterization techniques

Fourier-transform infrared (FTIR) spectroscopy were recorded from KBr pellets in the range 4000−400 cm^{-1} on a Shimadzu IR435 spectrometer.

Powder X-ray diffraction (PXRD) patterns were collected using a Rigaku Smartlab powder diffractometer at 45 kV, 200 mA for Cu Kα, (λ = 1.5406 Å) with a scan speed of 0.20 sec/step from 4.0 to 40º at a step size of 0.02º. The data were analyzed using the the Rigaku Powder Analysis Software package. The simulated powder patterns were calculated using Mercury software[1] based on single crystal diffraction data of the corresponding MOFs.

Thermogravimetric analyses (TGA) were performed using a NETZSCH leading thermal analysis system. All TGA experiments were performed under a N_2 atmosphere from ~20 °C to 600 °C at a rate of 2 °C /min.

Scanning electron microscopy-energy dispersive spectroscopy (SEM-EDS) was conducted using a Hitachi Regulus 8100 scanning electron microscope equipped with Bruker XFLASH 6160 X-EDS.

Elemental analyses (EA) were performed by A FLASH EA 1112 analyzer.

Raman spectroscopy was conducted using an inVia Raman microscope (RENISHAW Company) with a 532 nm laser and an 1800 lines/mm grating. The acquisition time was 10 s and accumulated for 5 cycles.

X-ray photoelectron spectroscopy (XPS) was carried out with an analysis area of 300 × 700 μm^2 and a pass energy of 30 eV. Spectra have been corrected to the main line of the carbon 1s spectrum (adventitious carbon) set to 284.8 eV. Spectra were analyzed using CasaXPS software (version 2.3.14) and collected using a Thermo Scientific ESCALAB 250Xi X-ray photoelectron spectrometer.

UV−vis spectroscopy were recorded on a METASH UV-8000 spectrometer.

Single Crystal X-ray diffraction (SCXRD) data for ALP-MOF-1·2.97I_2 and ALP-MOF-2·2H_2O were collected on a Bruker X8 Prospector Ultra equipped with an Apex II CCD detector and an IμS micro-focus CuK\α X-ray source (λ = 1.54178 Å) under N_2 stream at 220 K. Data for ALP-MOF-2·0.267I_3^- was collected on a Bruker X8 Prospector Ultra equipped with an Apex II CCD detector and an IμS micro-focus MoK\α X-ray source (λ = 0.71073 Å) under N_2 stream at 110 K. Indexing was performed using APEXII (Difference Vectors method). After integration of the data by Bruker program SAINT,[2] empirical absorption correction was applied using program SADABS.[3] The structures were solved by direct methods (ShelXT),[4] and non-hydrogen atoms were refined anisotropically by a least-squares fit on F2 using ShelXL on the OLEX 2.[5,6]

Section 1 (Crystallography) References

1. Macrae, C. F., Edgington, P. R., McCabe, P., Pidcock, E., Shields, G. P.,Taylor, R., Towler, M. & van de Streek, J. Mercury: visualization and analysis of crystal structures. *J. Appl. Cryst.* **2006**, *39*, 453−457.

2. SAINT (Bruker AXS, 2014).

3. SADABS (University of Gottingen, 2008).

4. Sheldrick, G. SHELXT-Integrated space-group and crystalstructure determination. *Acta Crystallogr., Sect. A: Found. Adv*. **2015**, *71*, 3−8.

5. Dolomanov, O. V.; Bourhis, L. J.; Gildea, R. J.; Howard, J. A. K.; Puschmann, H. OLEX2: a complete structure solution, refinement and analysis program. *J. Appl. Crystallogr.* **2009**, *42*, 339−341.

6. Sheldrick, G. Crystal structure refinement with SHELXL. *Acta Crystallogr., Sect. C: Struct. Chem*. **2015**, *71*, 3−8.

2. Iodine Adsorption Studies

2.1 Characterizations

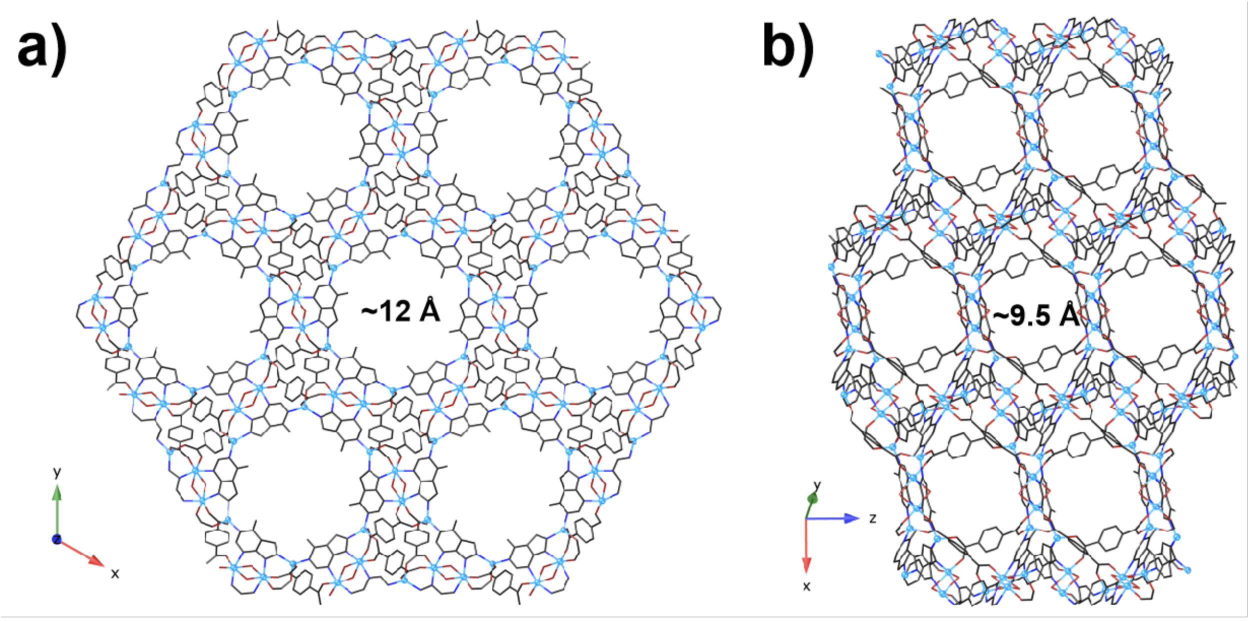

Figure S1. ALP-MOF-1/2 has two micropores, one roughly circular with a diameter of ~12 Å along the *c* crystallographic axis (a) and another lying along an axis nearly perpendicular to the main channel, which is roughly rectangular with a diameter of ~9.5 Å (b). Reproduced from Reference 1 (see Section 2 references). Copyright 2022 American Chemical Society.

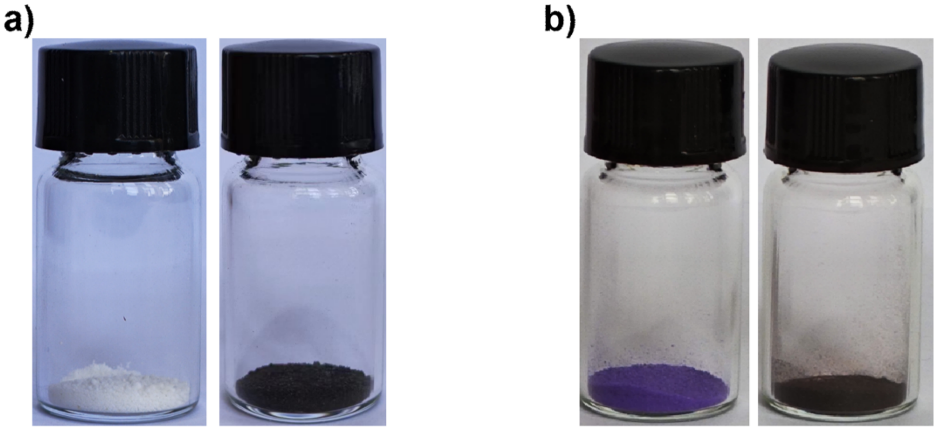

Figure S2. Color of samples: a) activated ALP-MOF-1 (left), ALP-MOF-1·2.97I_2 (right). b) activated ALP-MOF-2 (left), ALP-MOF-2·0.267I_3^- (right).

a)

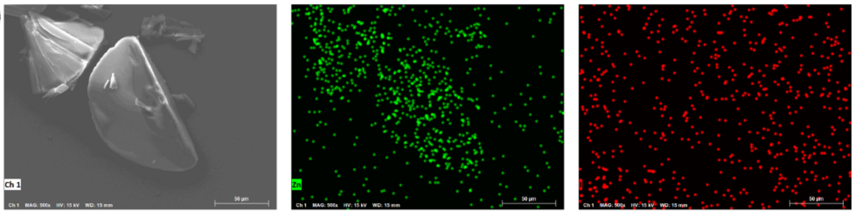

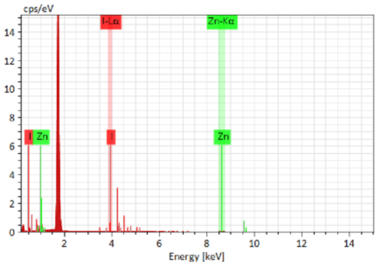

Element	Atomic Number	Weight [%]	Atomic [%]
Zn	30	99.17035729	99.57078443
I	53	0.82964271	0.429215569
		100	100

b)

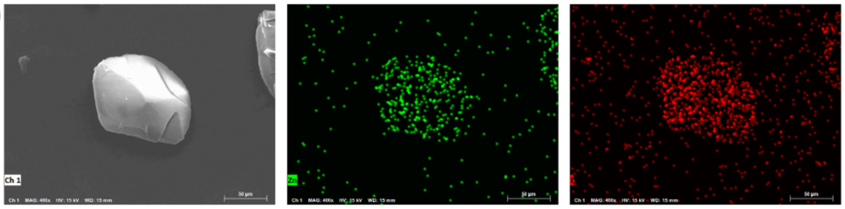

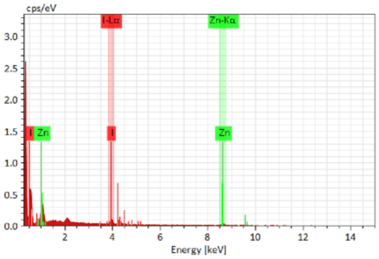

Element	Atomic Number	Weight [%]	Atomic [%]
Zn	30	41.27229457	26.5849117
I	53	58.72770543	73.4150883
		100	100

c)

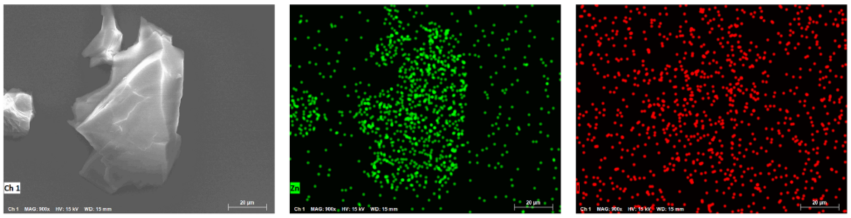

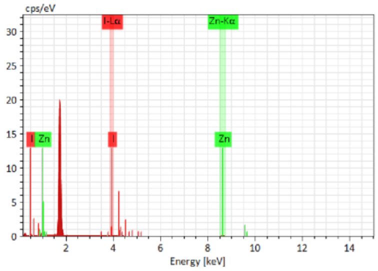

Element	Atomic Number	Weight [%]	Atomic [%]
Zn	30	96.22833544	98.02038468
I	53	3.771664562	1.979615317
		100	100

Figure S3. SEM images and EDS-mapping data for a) ALP-MOF-1, b) ALP-MOF-1·2.97I_2 and c) regenerated ALP-MOF-1 after ethanol rinse.

a)

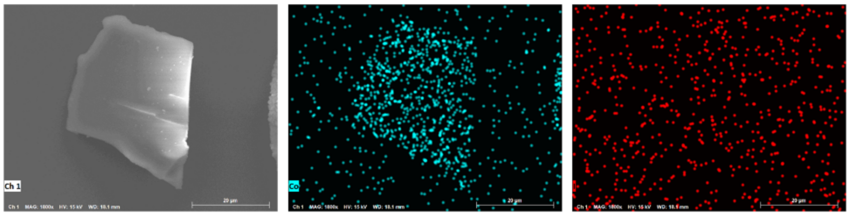

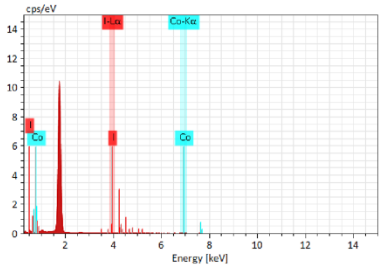

Element	Atomic Number	Weight [%]	Atomic [%]
Co	27	96.694324	98.43720654
I	53	3.305675997	1.562793458
		100	100

b)

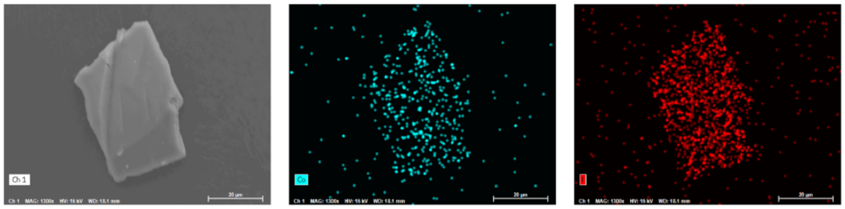

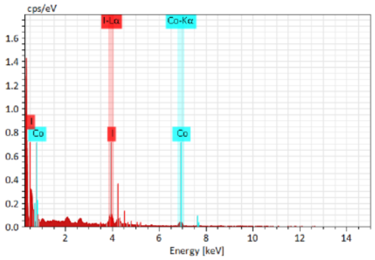

Element	Atomic Number	Weight [%]	Atomic [%]
Co	27	40.00909119	58.9510919
I	53	59.99090881	41.0489081
		100	100

c)

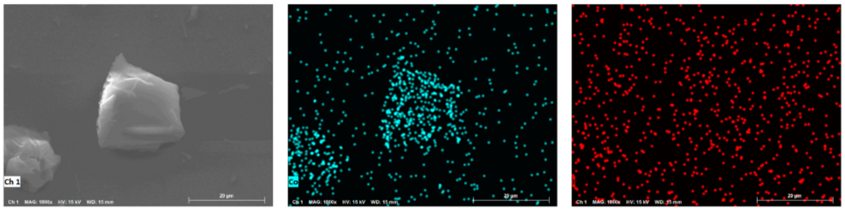

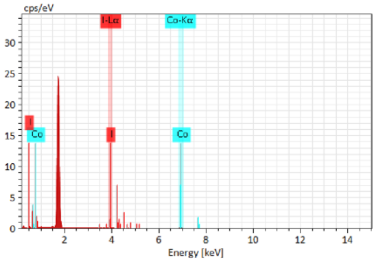

Element	Atomic Number	Weight [%]	Atomic [%]
Co	27	96.92424566	98.54772514
I	53	3.075754335	1.452274861
		100	100

Figure S4. SEM images and EDS-mapping data for a) ALP-MOF-2, b) ALP-MOF-2·0.267I_3^- and c) regenerated ALP-MOF-2 by rinsing ALP-MOF-2·0.167I_3^-·0.833I_5^-·0.167I_2 in ethanol.

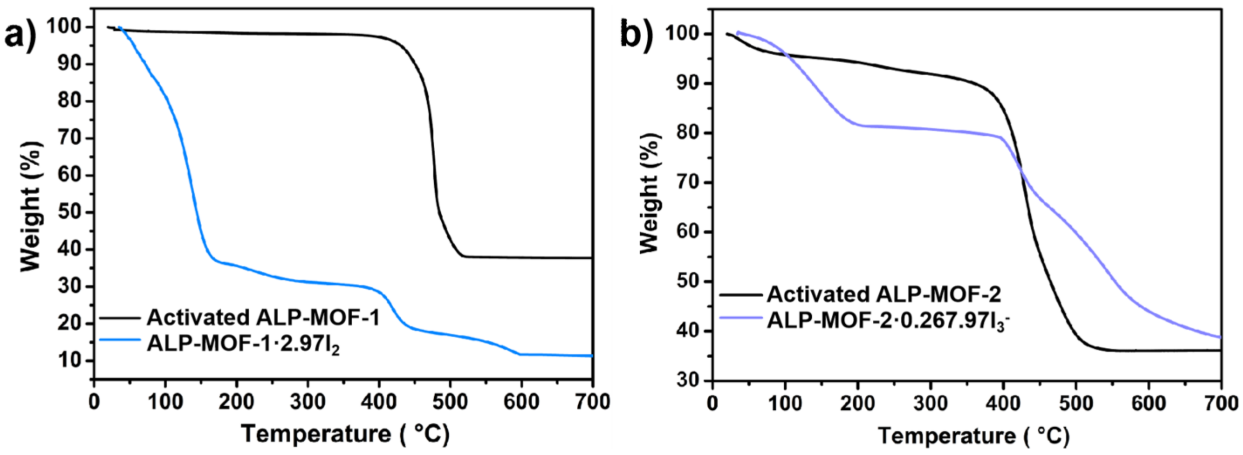

Figure S5. a) TGA of ALP-MOF-1 and ALP-MOF-1·2.97I_2. The initial weight loss of ~63% before 170 ºC corresponds to 2.97I_2. b) TGA of ALP-MOF-2 and ALP-MOF-2·0.267I_3^-. The initial weight loss of ~18% before 200 ºC corresponds to 1 H_2O and 0.267 I_2 decomposed from I_3^- under heating. Subsequent weight loss steps are ascribed to sample decomposition.

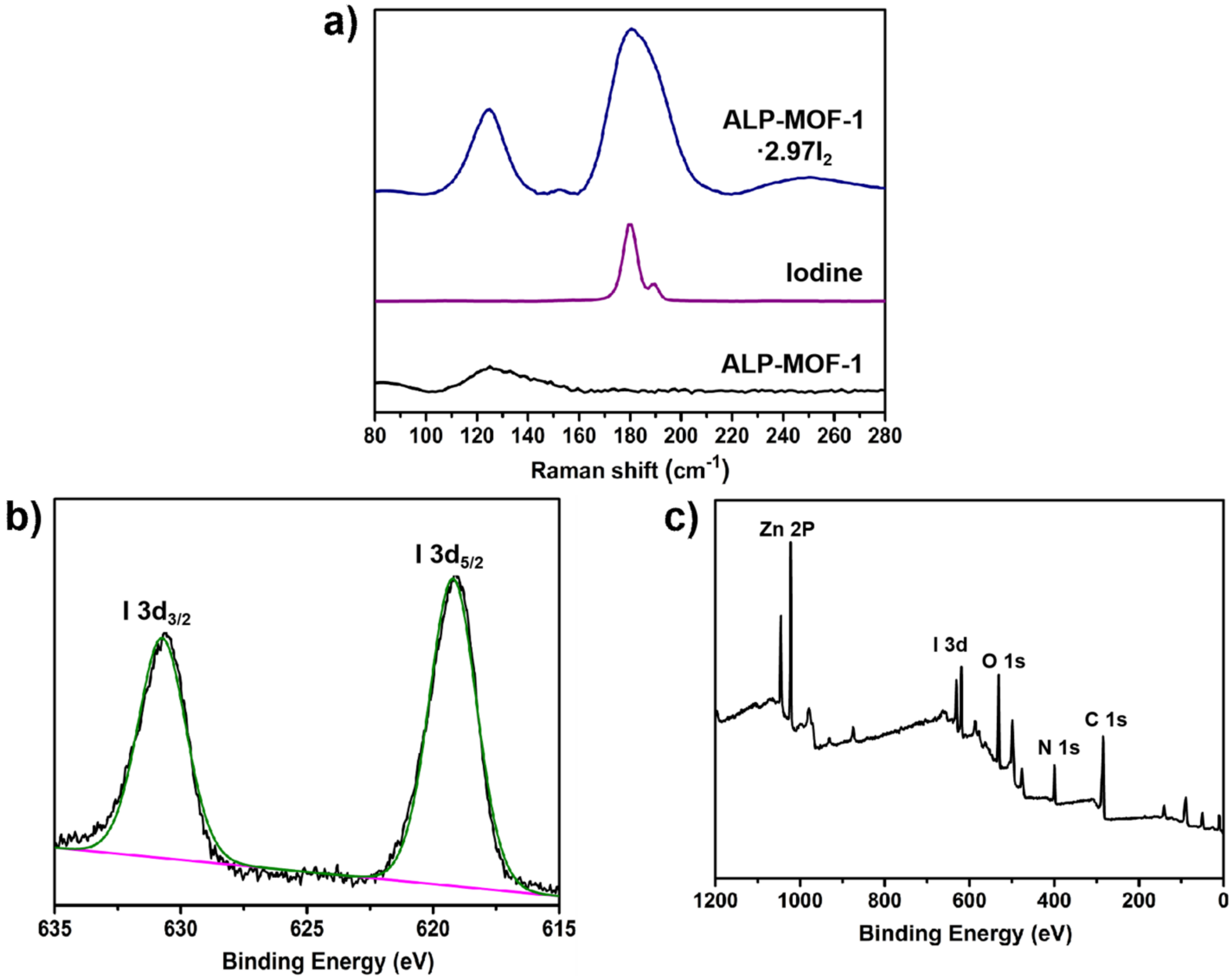

Figure S6. a) Comparison of Raman spectra of ALP-MOF-1, solid iodine, and ALP-MOF-1·2.97I_2. b) The I 3d XPS of ALP-MOF-1·2.97I_2. c) Full XPS spectra of ALP-MOF-1·2.97I_2. Spectra have been corrected to the main line of the carbon 1s spectrum (adventitious carbon) set to 284.8 eV.

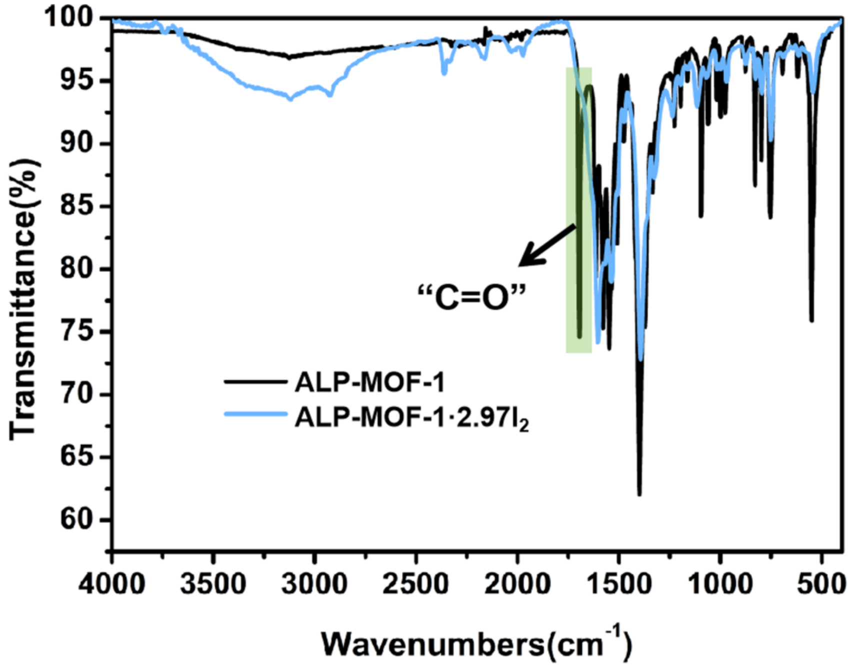

Figure S7. FT-IR spectra of ALP-MOF-1 and ALP-MOF-1·2.97I_2.

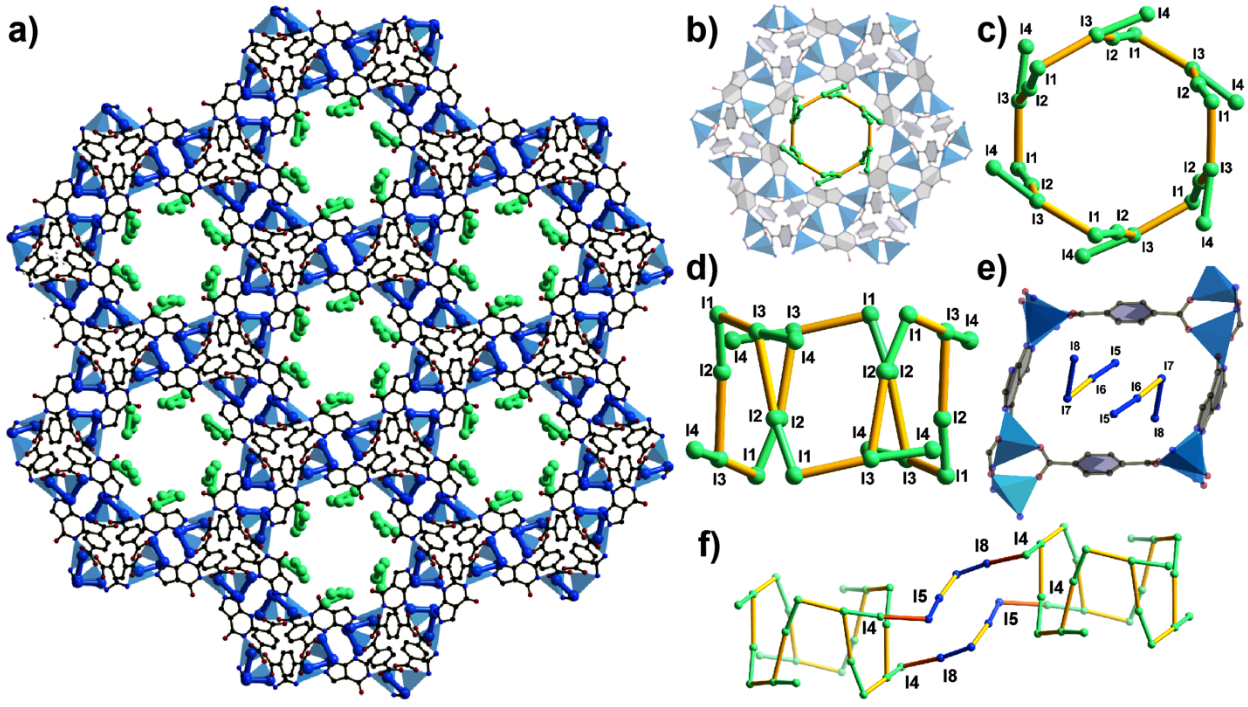

Figure S8. a) Crystallographic visualization of adsorbed iodine molecules confined in ALP-MOF-1·2.97I_2. b) I24 cluster lies in the larger pore along the *c* crystallographic direction. c) Top and d) side views of alternating six groups of I_1−I_2 and I_3−I_4 forming the I24 cluster via I_1···I_3 and I_2···I_3 interactions. e) L-shaped I4 bridges sitting in the smaller pores consist of I_5−I_6 and I_7−I_8 through I_6···I_7 interactions. f) 6-connected cylinder-shaped I24 clusters are connected by double L-shaped I4 bridges through strong inter-iodine molecular interactions (I_4···I_5, I_4···I_8).

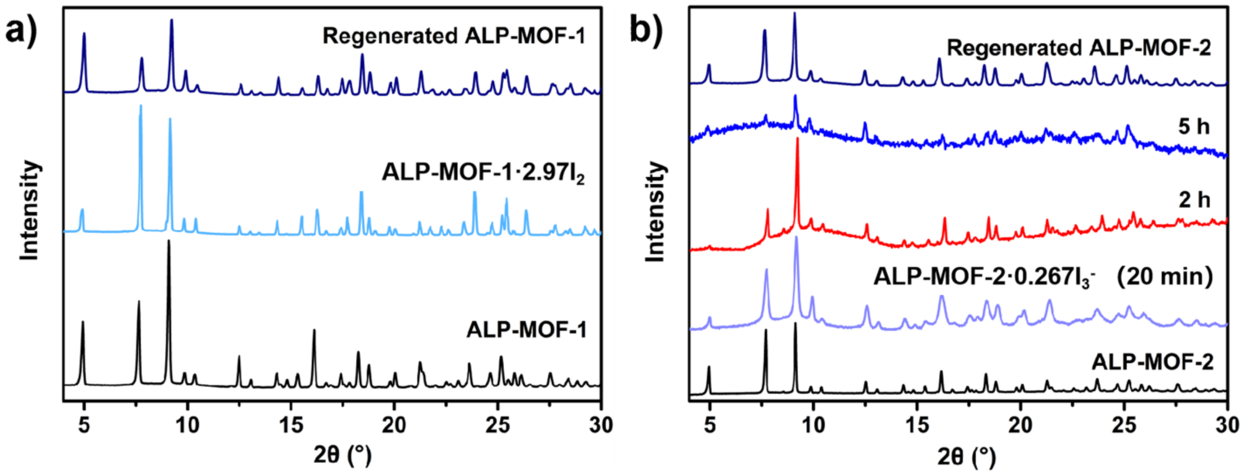

Figure S9. PXRD patterns for a) ALP-MOF-1, ALP-MOF-1·2.97I_2, and regenerated ALP-MOF-1 after ethanol rinse, and b) ALP-MOF-2, ALP-MOF-2 after exposure to iodine vapor as a function of time and regenerated ALP-MOF-2 after rinsing ALP-MOF-2·0.167I_3^-·0.833I_5^-·0.167I_2 (sample exposed to iodine for 5 h) in ethanol.

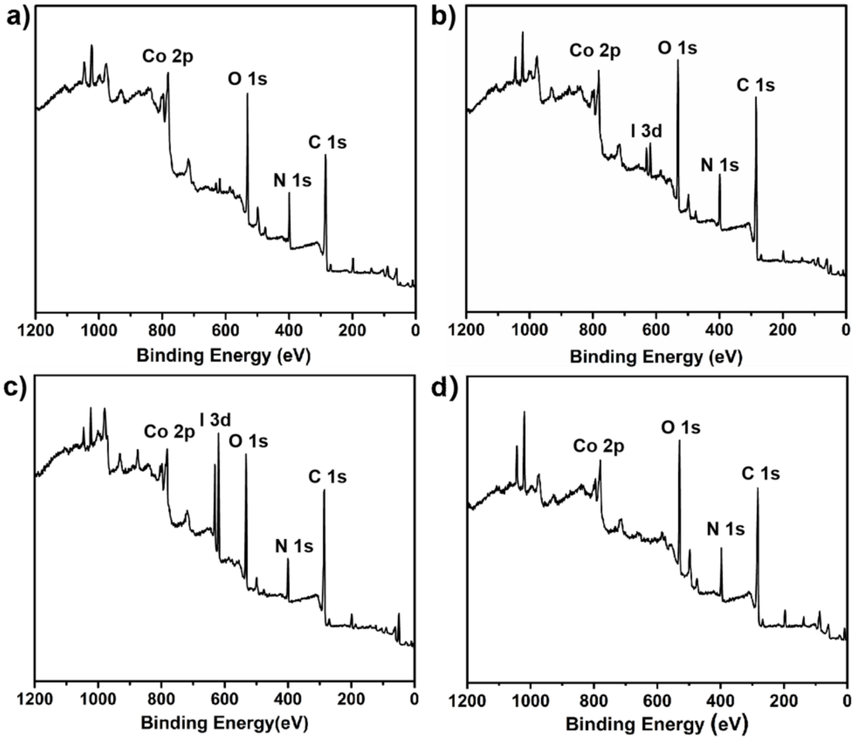

Figure S10. Full XPS spectra of a) ALP-MOF-2, b) ALP-MOF-2·$0.2I_3^-$·$0.6I_5^-$, c) ALP-MOF-2·$0.167I_3^-$·$0.833I_5^-$·$0.167I_2$ and d) regenerated ALP-MOF-2. Spectra have been corrected to the main line of the carbon 1s spectrum (adventitious carbon) set to 284.8 eV.

Table S1. Summary of formulation and adsorption capacities for iodine-loaded samples of ALP-MOF-2.

**Molecular Formula**	**Iodine Exposure Time**	**Co^{III}:Co^{II} Ratio** (from XPS)	**Adsorption capacity** (based on determined formulae)	**Adsorption capacity** (based on gravimetric study)	**Crystalline** (Yes or No)
[$Co^{III}_{0.267}Co^{II}_{1.733}$($\mu_2$-$H_2O$)(ALP)(BDC)]·$0.267I_3^-$<br>ALP-MOF-2·$0.267I_3^-$	20 min	~ 1:6.5	~ 23.4 wt%	~ 24.6 wt%	Yes
[$Co^{III}_{0.8}Co^{II}_{1.2}$($\mu_2$-$H_2O$)(ALP)(BDC)]·$0.2I_3^-$·$0.6I_5^-$<br>ALP-MOF-2·$0.2I_3^-$·$0.6I_5^-$	2 h	~ 1:1.5	~ 105 wt%	~ 102 wt%	Yes
[$Co^{III}Co^{II}$(μ_2-<br>H_2O)(ALP)(BDC)]·$0.167I_3^-$·$0.833I_5^-$·$0.167I_2$<br>ALP-MOF-2·$0.167I_3^-$·$0.833I_5^-$·$0.167I_2$	5 h	~ 1:1	~ 146 wt%	~ 150 wt%	No

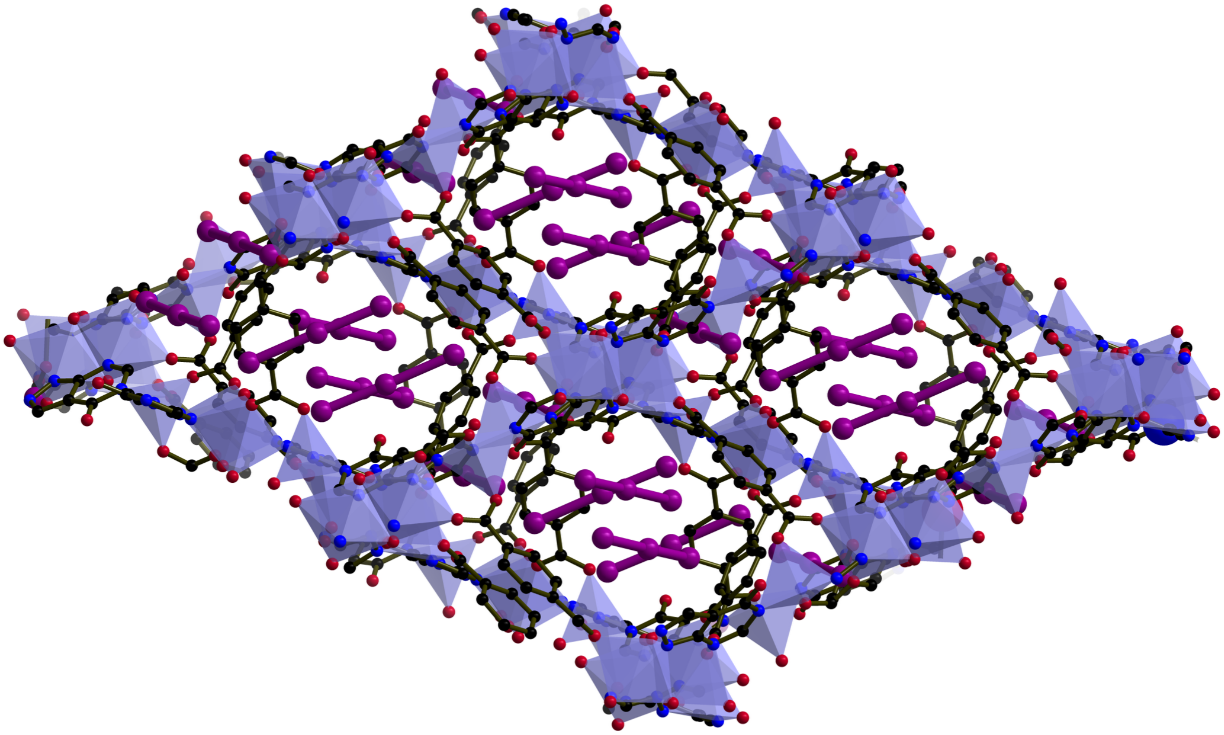

Figure S11. I_3^- units are located in the smaller pores of ALP-MOF-2·0.267I_3^- along the *b* crystallographic direction.

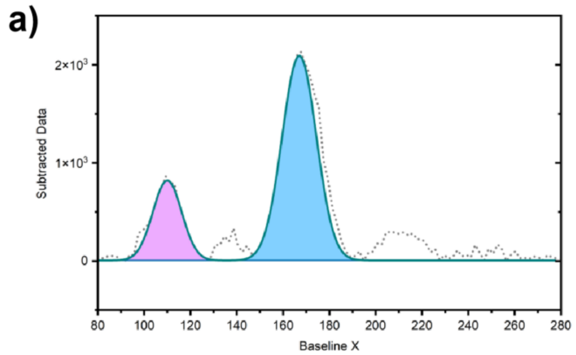

Fitting Results

Peak Index	Peak Type	Area Intg	FWHM	Max Height	Center Grvty	Area IntgP
1	Gaussian	12738.99621	14.66718	815.93656	110	24.75388
2	Gaussian	38723.63151	17.41519	2088.88986	167	75.24612

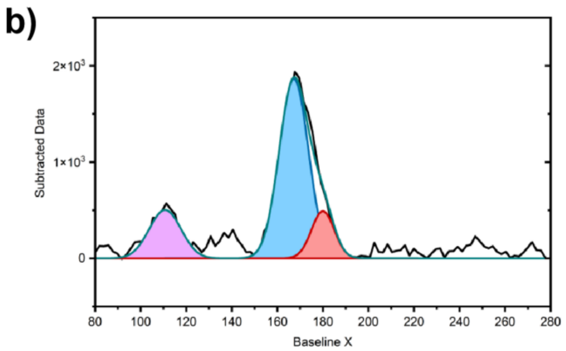

Fitting Results

Peak Index	Peak Type	Area Intg	FWHM	Max Height	Center Grvty	Area IntgP
1	Gaussian	5616.78438	11.32075	466.10121	110.54609	14.52948
2	Gaussian	27820.45056	14.02173	1863.9338	167.53268	71.96584
3	Gaussian	5220.62232	10.11221	485.0023	180	13.50469

Figure S12. The fitting and integration results of Raman spectra for determining the ratios of I_3^-, I_5^-, and I_2.

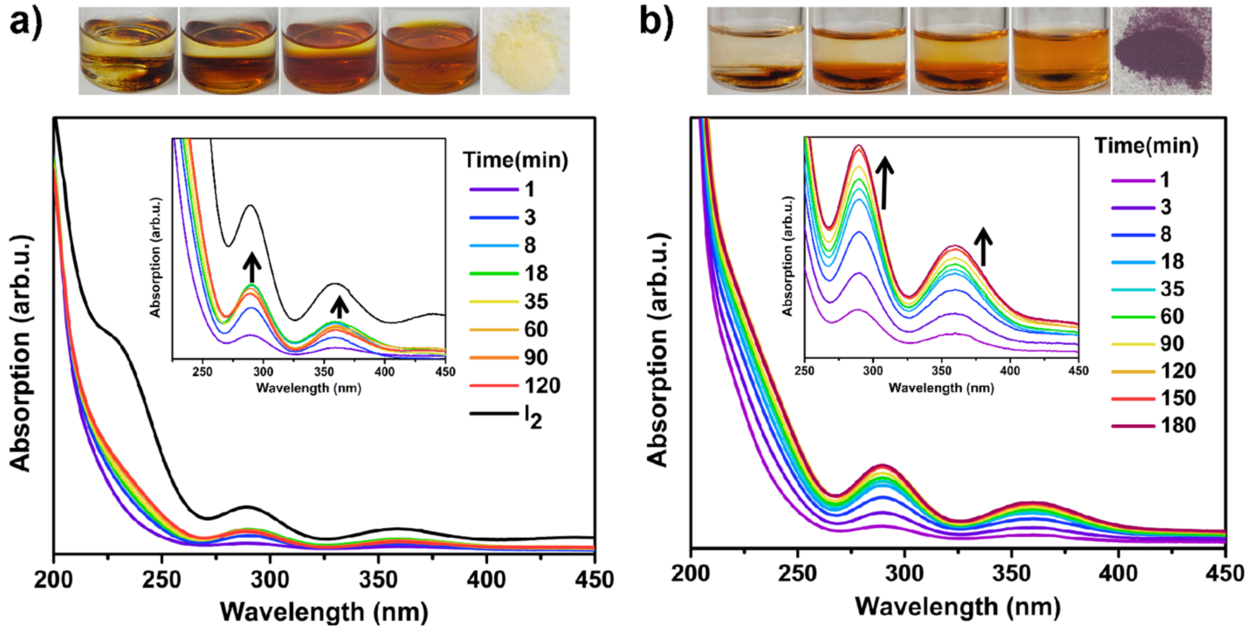

Figure S13. Iodine release from ALP-MOF-1 (a) and ALP-MOF-2 (b). The photographs on the extreme left and extreme right in (a) or (b) indicate the regenerated products and color changes during soaking ~15 mg of ALP-MOF-1·2.97I_2 and ALP-MOF-2·0.167I_3^-·0.833I_5^-·0.167I_2 in 5 mL ethanol, respectively. The photos of the vials show progressive release of iodine into ethanol over time. a) Time-dependent UV/vis spectra of ALP-MOF-1·2.97I_2 show λ_{max} at 288 and 360 nm, corresponding to polyiodide I_3^-, which are generally stabilized by H^+ and obtained from the reaction I_2 with decomposed I^-. b) Time-dependent UV/vis spectra of ALP-MOF-2·0.167I_3^-·0.833I_5^-·0.167I_2 show the release of I_3^- after ethanol rinse (No detection of I_5^- is possibly due to the decomposition of I_5^- to I_3^- and I_2 in the presence of ethanol). Notes: the pink color of regenerated ALP-MOF-2 is attributed to the capture of water molecules from ethanol, which is confirmed by SCXRD (Figure S14).

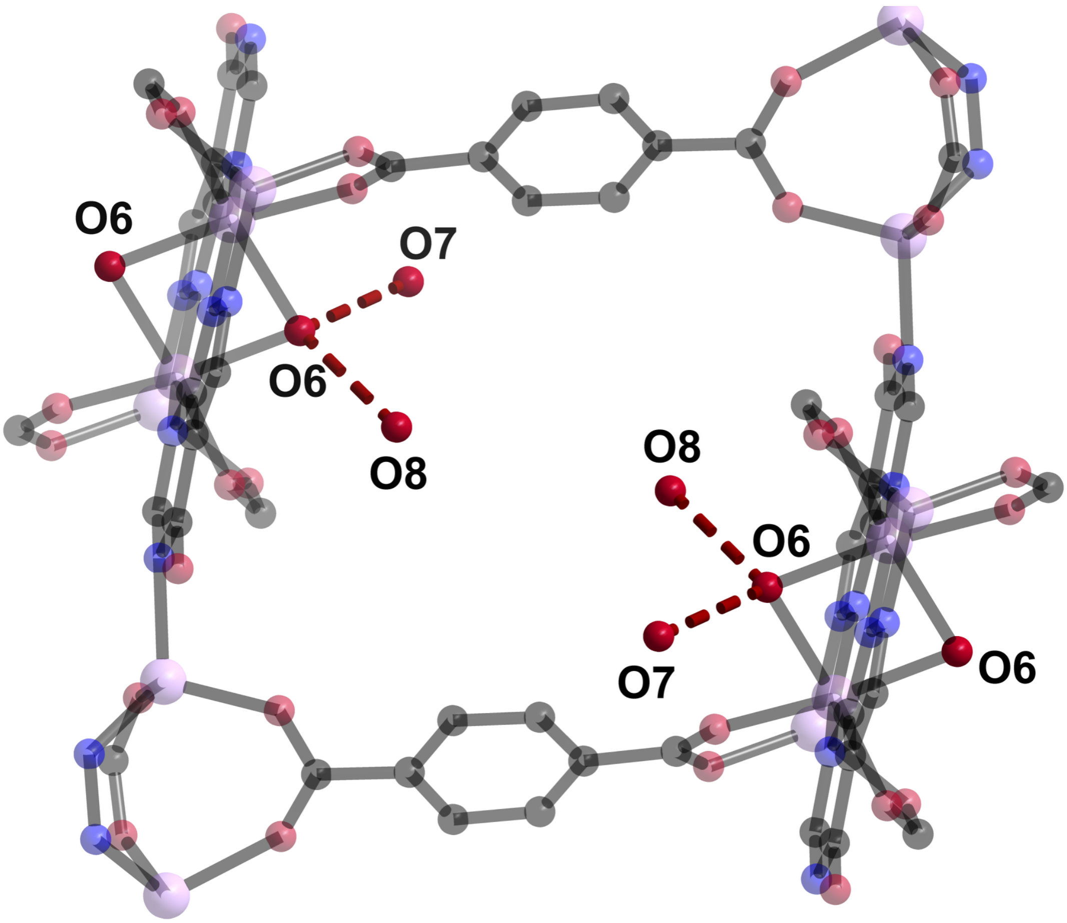

Figure S14. Single crystal structure of regenerated ALP-MOF-2 (ALP-MOF-2·2H_2O). Adsorbed water molecules O7 and O8 located in the smaller pore are hydrogen bonded with the bridging water O6 with the O···O distances of O6···O7 = 2.641 Å and O6···O8 = 2.672 Å (Hydrogen atoms are omitted for clarity).

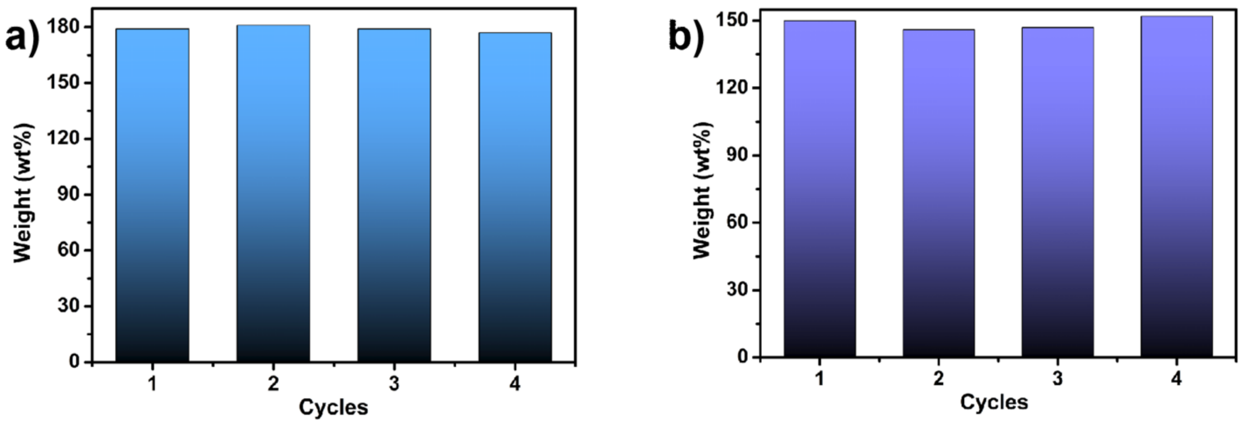

Figure S15. Regenerated ALP-MOF-1 and ALP-MOF-2 almost maintain the iodine adsorption capacities after four cycles.

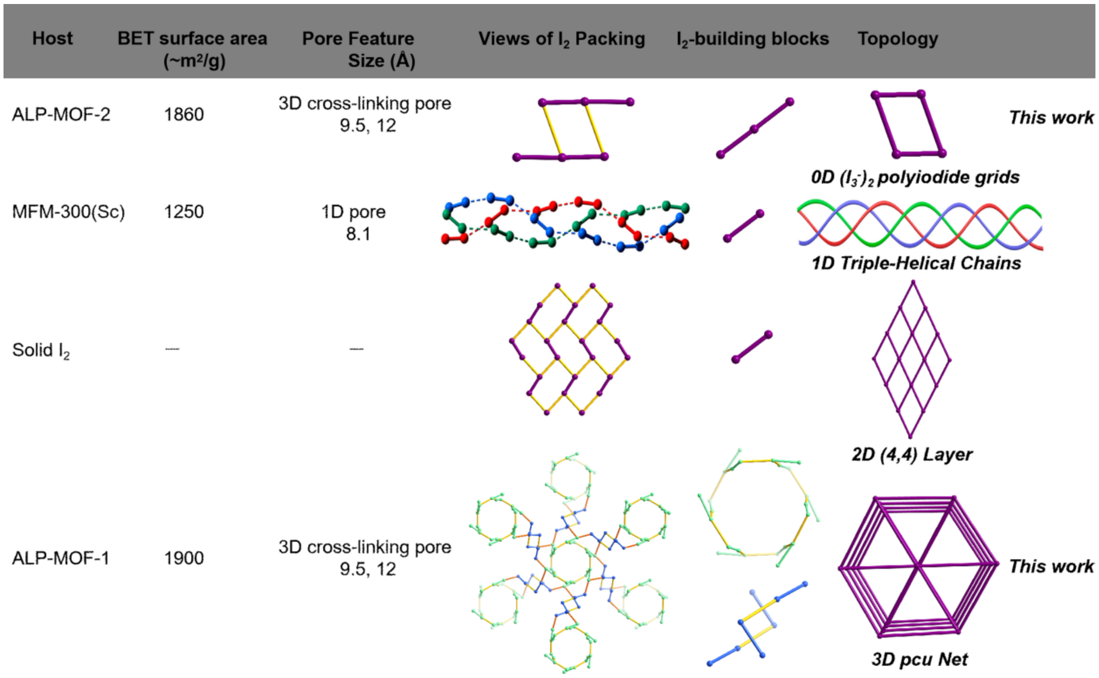

Figure S16. Illustration of the usual 2D solid iodine framework and selected unusual iodine aggregations confined in MOFs, ranging from 0D grid, 1D triple-helical chain to 3D pcu network (All the colored spheres represent iodine atoms in the columns of **views of I_2 packing** and **I_2-buiding blocks**).

Table S2. I_2 absorptive capabilities in reported MOFs within 5h at ambient pressure via vapor diffusion.

MOFs	BET surface area (m^2 /g)	Iodine uptake (~wt%)	Temperature (℃)	ref
**ALP-MOF-1**	**1900**	**179**	**50**	**This work**
**ALP-MOF-2**	**1860**	**150**	**50**	**This work**
MFM-300(Sc)	1250	< 154	70	2
MFM-170	2408	110	80	3
MFM-174	2251	110	80	3
NJU-Bai-20	2081	110	80	3
$[(ZnI_2)_3(TPT)_2]\cdot5.5(C_6H_5NO_2)$	-	< 66	RT	4
SBMOF-1	145	< 20	RT	5
SBMOF-2	195	< 30	RT	5
UPC-158	2170	178	70	6
UPC-158-HF	2137	219	70	6
UPC-158-HCl	2289	292	70	6
UPC-158-HBr	2151	275	70	6
UPC-158-HI	1954	259	70	6
MOF-808	1930	120	80	7
NU-1000	2126	75	80	7
UiO-66	1072	35	80	7
MOF-867	2404	50	80	7
UiO-66	2638	25	80	7
Zn_2(tptc)(apy)	168	50	75	8

2.2 Identifications of I···I interactions

R−X···X−R interactions (X = halogen atom) can occur preferentially according to two different geometries, which are classified as type I (symmetrical interactions where $\theta_1 = \theta_2$) and type II (bent interactions where $\theta_1 \approx 180°$ and $\theta_2 \approx 90°$).[9] Type I interactions ($0° \leq |\theta_1 - \theta_2| \leq 15°$) are geometric contacts that originate from required close-packing, which are not halogen bonds (XBs) according to the IUPAC definition. While Type II interactions ($30° \leq |\theta_1 - \theta_2| \leq 105°$) are true XBs, which originate from the pairing between the electrophilic area on one halogen atom (X1) and the electrophilic area on the other X2.[10] Type II interactions are found to be most favored in iodinated derivatives.[11]

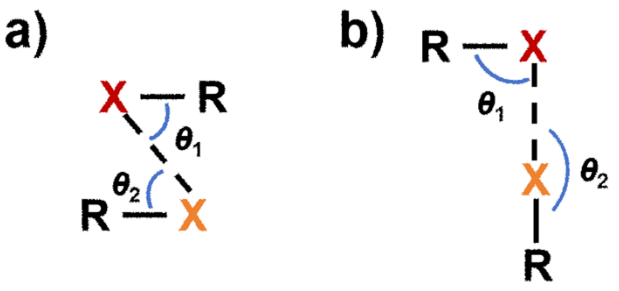

Figure S17. Structural scheme for type I (a) and type II (b) halogen···halogen short contacts (X = halogen atom, and R = halogen atom, C, N, O, etc.). Type II contacts are XBs.

Apart from the angle, another key factor for identifying the formed XBs within iodine or polyiodide networks is the I···I distance, which is considered to be significantly shorter than the sum of van der Waals radii of iodine (3.96 Å).

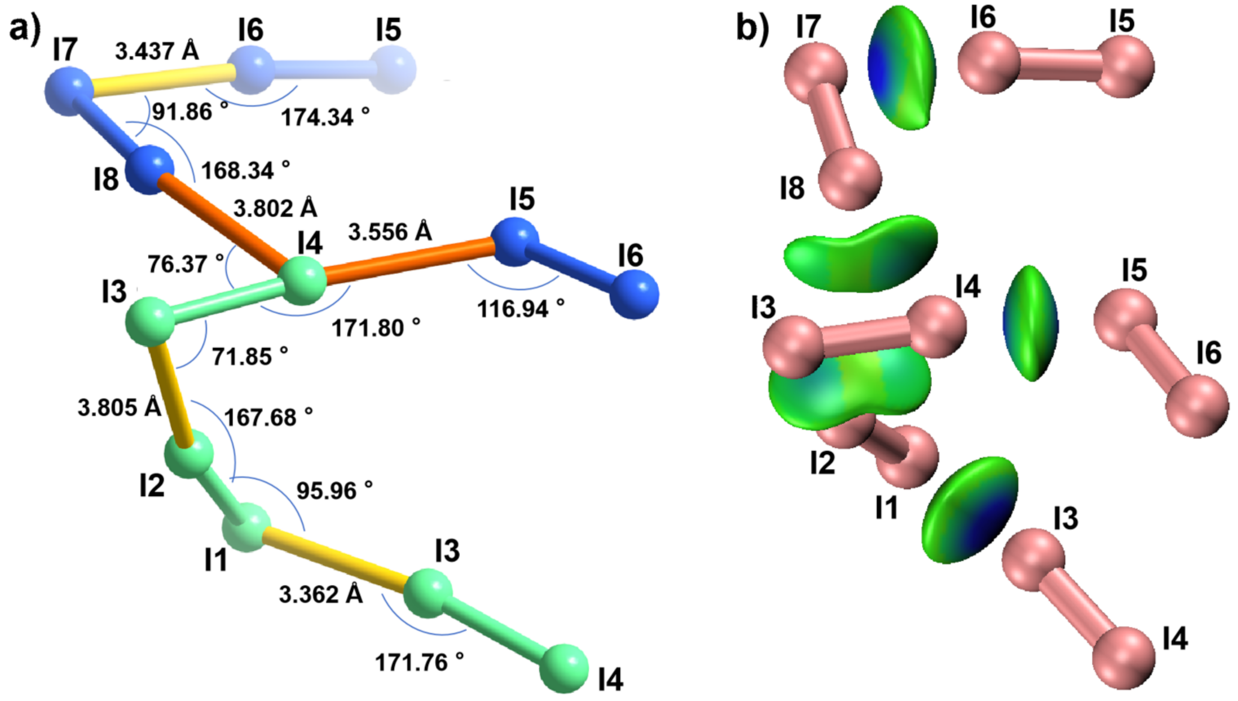

Figure S18. (a) The sum of the distances and angles of I-I⋯I-I interactions arising from SCXRD data of ALP-MOF-1·2.97I_2 (Golden and orange solid lines represent I⋯I interactions). (b) The strength of theoretical XBs well matches with the IGM studies.

Table S3. The sum of the distances and $|\theta_1-\theta_2|$ of I-I⋯I-I interactions in ALP-MOF-1·2.97I_2 for the identifications of XBs.

	**Distance (Å)**	**$|\theta_1-\theta_2|$ (°)**	**Type interactions**	**XBs**
I_2-I_1⋯I_3-I_4	3.363	75.80	II	Strong XBs
I_1-I_2⋯I_3-I_4	3.805	95.83	II	Weak XBs
I_3-I_4⋯I_5-I_6	3.556	54.86	II	Medium XBs
I_3-I_4⋯I_8-I_7	3.802	91.97	II	Weak XBs
I_8-I_7⋯I_6-I_5	3.437	82.48	II	Strong XBs

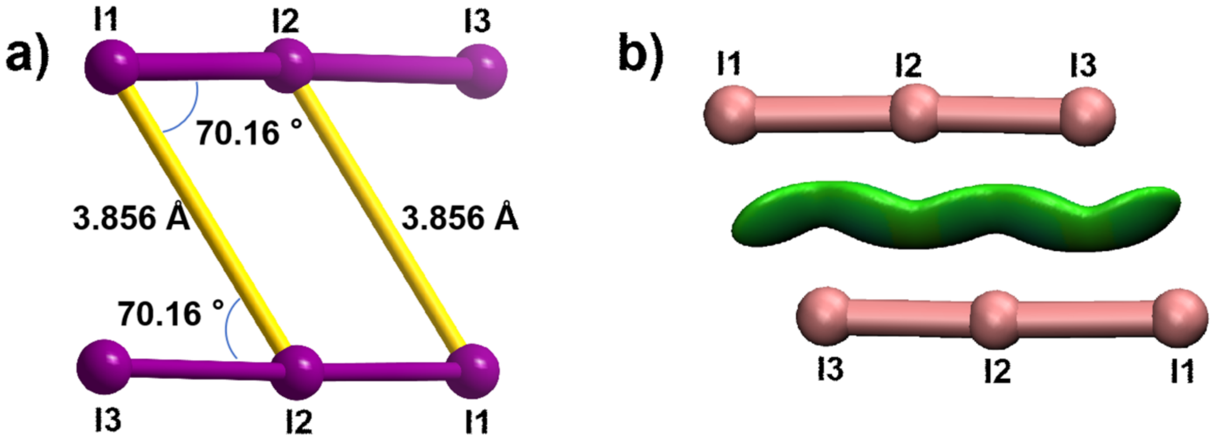

Figure S19. (a) The distances and angles of I-I-I···I arising from SCXRD data of ALP-MOF-2·0.267I_3^- indicates I···I interactions are type I interactions, not XBs (Golden solid lines represent I···I interactions). (b) The strength of I···I matches with the IGM studies.

Section 2 References

1. Han, Y.; Das, P.; He, Y.; Sorescu, D. C.; Jordan, K. D.; Rosi, N. L. Crystallographic mapping and tuning of water adsorption in metal−organic frameworks featuring distinct open metal sites. *J. Am. Chem. Soc.* **2022**, *144*, 19567−19575.

2. Zhang, X., Silva, I., Godfrey, H., Callear, S., Sapchenko, S., Cheng, Y., Vitorica-Yrezabal, I., Frogley, M., Cinque, G., Tang, C., Giacobbe, C., Dejoie, C., Rudic, S., Ramirez-Cuesta, A., Denecke, M., Yang, S. Schröder, M. Confinement of iodine molecules into triple-helical chains within robust metal-organic frameworks. *J. Am. Chem. Soc.* **2017**, *139*, 16289−16296.

3. Li, J.; Zhang, X.; Fan, M.; Chen, Y.; Ma, Y.; Smith, G. L.; Vitorica-yrezabal, I. J.; Lee, D.; Xu, S.; Schröder, M.; Yang, S. Direct observation of enhanced iodine binding within a series of functionalized metal-organic frameworks with exceptional irradiation stability. *J. Am. Chem. Soc.* **2024**, *146*, 14048−14057.

4. Brunet, G.; Safin, D.; Aghaji, M.; Robeyns, K.; Korobkov, I.; Woo, T.; Murugesu, M. Stepwise crystallographic visualization of dynamic guest binding in a nanoporous framework. *Chem. Sci.* **2017**, *8*, 3171−3317.

5. Banerjee, D., Chen, X., Lobanov, S., Plonka, A., Chan, X., Daly, J., Kim, T., Thallapally, P. & Parise, J. Iodine Adsorption in metal organic frameworks in the presence of humidity. ACS Appl. Mater. Interfaces **2018**, *10*, 10622−10626.

6. Guo, B., Li, F., Wang, C., Zhang, L. Sun, D. A rare (3,12)-connected zirconium metal-organic framework with efficient iodine adsorption capacity and pH sensing. *J. Mater. Chem.* A **2019**, *7*, 13173−13179.

7. Chen, P., He, X., Pang, M., Dong, X., Zhao, S. Zhang, W. Iodine capture using Zr-based metal-organic frameworks (Zr-MOFs): Adsorption performance and mechanism. *ACS Appl. Mater. Interfaces* **2020**, *12*, 20429–20439.

8. Yao, R. X., Cui, X., Jia, X. X., Zhang, F. Q. & Zhang, X. M. A luminescent zinc(II) metal-organic framework (MOF) with conjugated π -electron ligand for high iodine capture and nitro-explosive detection. *Inorg. Chem.* **2016**, *55*, 9270–9275.

9. Desiraju, G. R.; Parthasarathy, R. The Nature of Halogen··· halogen Interactions: Are Short Halogen Contacts due to Specific Attractive Forces or due to Close Packing of Nonspherical Atoms? *J. Am. Chem. Soc.* **1989**, *111*, 8725−8726.

10. Metrangolo, P.; Resnati, G. Metal-Bound Halogen Atoms in Crystal Engineering. *Chem. Commun.* **2013**, *49*, 1783−1785.

11. Mukherjee, A.; Tothadi, S.; Desiraju, G. R. Halogen Bonds in Crystal Engineering: Like Hydrogen Bonds yet Different. *Acc. Chem. Res.* **2014**, *47*, 2514−2524.

Section 3. Computational Methods

The spin-polarized density functional theory (DFT) calculations[1,2] were carried out in the CP2K code.[3] All calculations employed a mixed Gaussian and planewave basis sets. Core electrons were represented with norm-conserving Goedecker-Teter-Hutter pseudopotentials,[4-6] and the valence electron wavefunction was expanded in a double-zeta basis set with polarization functions[7] along with an auxiliary plane wave basis set with an energy cutoff of 450 eV. The generalized gradient approximation exchange-correlation functional of Perdew, Burke, and Enzerhof (PBE)[8] was used. Each configuration was optimized with the Broyden-Fletcher-Goldfarb-Shanno (BGFS) algorithm with SCF convergence criteria of 1.0×10^{-6} au. The van der Waals correction of Grimme's DFT-D3 model was also adopted[9].

The adsorption energy between the iodine species and the MOFs was calculated using the following equation:

$$\Delta E = E_{iodine\ species@\mathrm{MOF}} - E_{\mathrm{MOF}} - E_{\mathrm{iodine\ species}} \qquad \text{(S1)}$$

In Eq. (S1), $E_{\mathrm{iodine\ species@MOF}}$ and E_{MOF} represent the total energies of the MOF with and without the adsorption of iodine species, respectively. $E_{\mathrm{iodine\ species}}$ is the total energy of the iodine species. According to this equation, a negative adsorption energy corresponds to a stable adsorption structure.

Independent gradient model (IGM) analyses were adopted to visually understand the noncovalent interactions for the host-guest interaction[10]. In this work, IGM analyses were based on the DFT calculated iodine species adsorption configurations in MOFs, in which the MOF framework fragment was intercepted for clearly showing various interactions. The IGM analyses were achieved by Multiwfn software package 3.8[11], while the VMD 1.9.3 program[12] was used to render the IGM visualized isosurfaces of weak interaction for the host-guest interaction.

Section 3 References

1. Hohenberg, P. and Kohn, W. Inhomogeneous Electron Gas. *Physical Review.* **1964**, *136*, 864−871.

2. Kohn, W. and Sham, L. J. Self-Consistent Equations Including Exchange and Correlation Effects. *Physical Review.* **1965**, *140*, 1133−1138.

3. VandeVondele, J.; Krack, M.; Mohamed, F.; Parrinello, M.; Chassaing, T.; Hutter, J. Quickstep: Fast and accurate density functional calculations using a mixed Gaussian and plane waves approach. *Comput. Phys. Commun.* **2005**, *167*, 103−128.

4. Goedecker, S.; Teter, M.; Hutter, J. Separable Dual-Space Gaussian Pseudopotentials. *Phys. Rev. B.* **1996**, *54*, 1703−1710.

5. Hartwigsen, C.; Goedecker, S.; Hutter, J. Relativistic Separable Dual-Space Gaussian Pseudopotentials from H to Rn. *Phys. Rev. B.* **1998**, *58*, 3641−3662.

6. Krack, M.; Parrinello, M. All-electron ab-initio Molecular Dynamics. *Phys. Chem. Chem. Phys.* **2000**, *2*, 2105−2112.

7. VandeVondele, J.; Hutter, J. Gaussian Basis Sets for Accurate Calculations on Molecular Systems in Gas and Condensed Phases. *J. Chem. Phys.* **2007**, *127*, 114105.

8. Perdew, J. P.; Burke, K.; Ernzerhof, M. Generalized gradient approximation made simple. *Phys. Rev. Lett.* **1996**, *77*, 3865−3868.

9. Grimme, S.; Antony, J.; Ehrlich, S.; Krieg, H. A consistent and accurate *ab initio* parametrization of density functional dispersion correction (DFT-D) for the 94 elements H-Pu. *J Chem Phys.* **2010**, *132*, 154104.

10. Lefebvre, C.; Rubez, G.; Khartabil, H.; Boisson, J. C.; Contreras-Garcia, J.; Henon, E. Accurately extracting the signature of intermolecular interactions present in the NCI plot of the reduced density gradient versus electron density. *Phys. Chem. Chem. Phys.* **2017**, *19*, 17928−17936.

11. Lu, T.; Chen, F. W; Multiwfn: A multifunctional wave function analyzer. *J. Comput. Chem.* **2012**, *33*, 580−592.

12. Humphrey, W.; Dalke, A.; Schulten, K. VMD: Visual molecular dynamics. *J. Mol. Graph.* **1996**, *14*, 33−38.

Section 4. Metal Ion Composition Characterization

Mixed Zn(II)/Co(II) ALP-MOFs were incubating as-synthesized ALP-MOF-1 single crystals (~30 mg) in a CH_3CN solution of $CoCl_2$ (20 mL, 0.01 M) at room temperature can afford analogues with the following Zn/Co ratios (note: incubation time in parentheses): 0.89:0.11 (～0.5 h), 0.58:0.42 (～12 h), 0.76:0.24 (～24 h). The Zn/Co ratios were determined using SEM-EDS on thoroughly ground samples of the MOF crystals. To ensure homogeneous sampling of the materials, all MOF samples were thoroughly ground prior to analysis using SEM-EDS and each Zn/Co ratio result is the average value of three analyses.

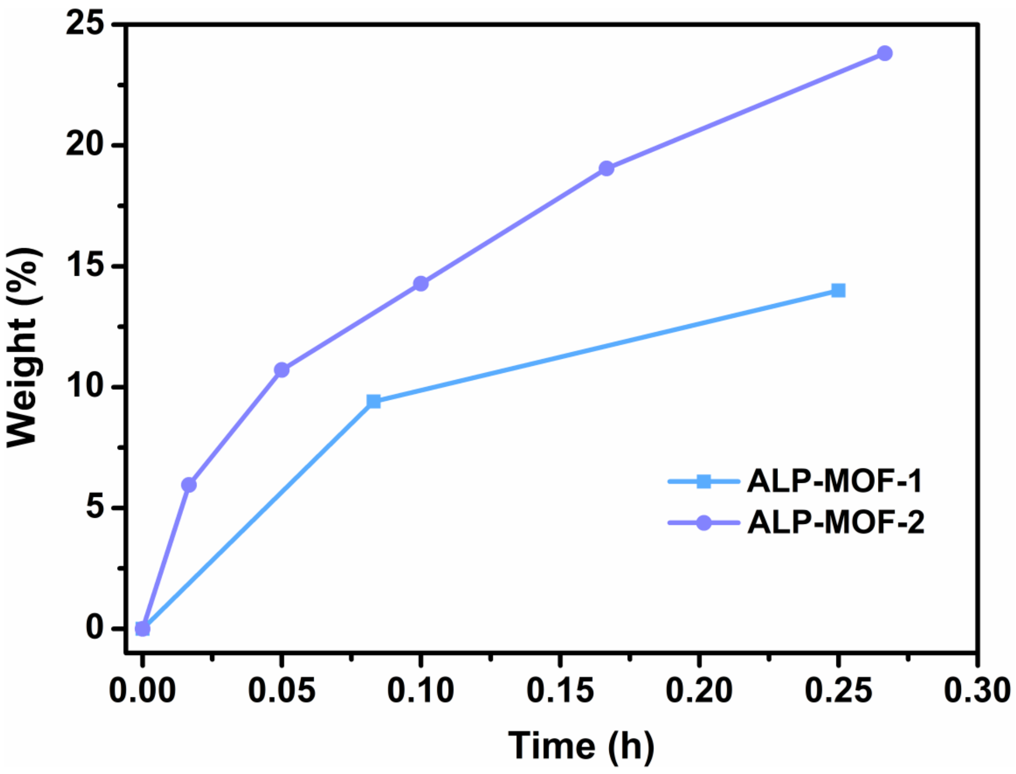

Figure S20. Localised iodine adsorption isotherms indicate a rapid I_2 adsorption at early times (< 0.3 h) below 25 wt% for ALP-MOF-1 vs ALP-MOF-2.

a)

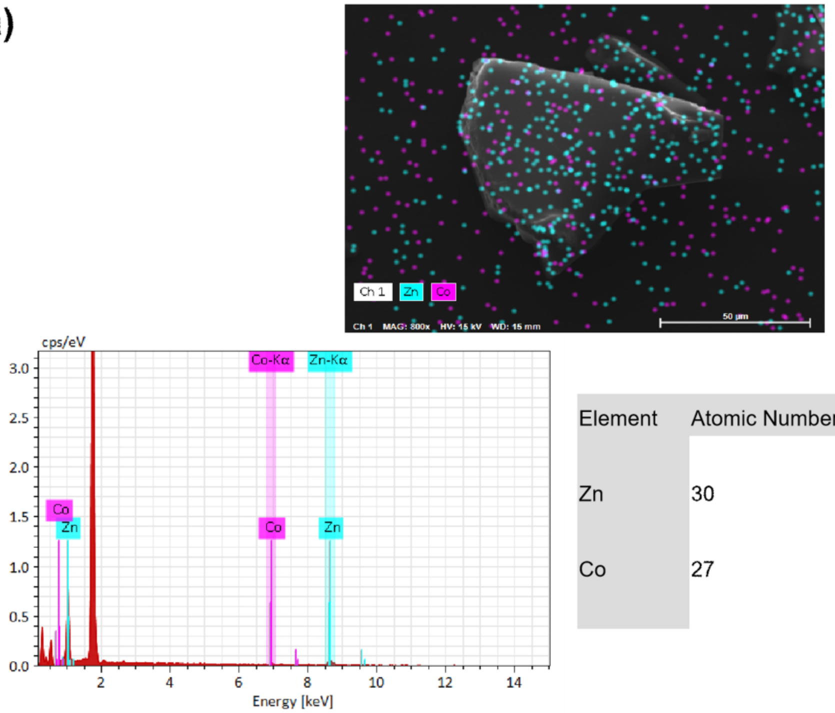

Element	Atomic Number	Weight [%]	Atomic [%]
Zn	30	91.19	88.62
Co	27	8.81	11.38
		100	100

b)

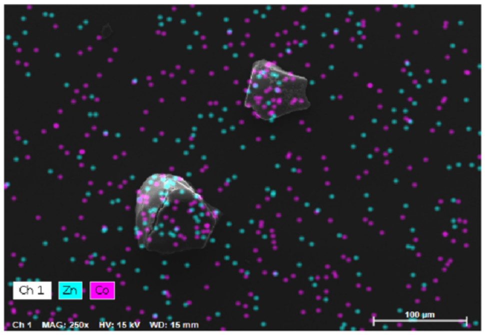

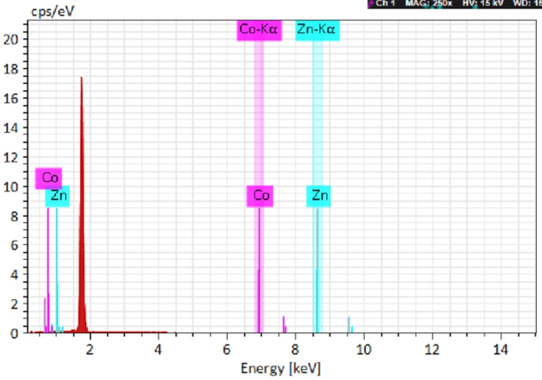

Element	Atomic Number	Weight [%]	Atomic [%]
Zn	30	61.17	58.38
Co	27	38.83	41.62
		100	100

c)

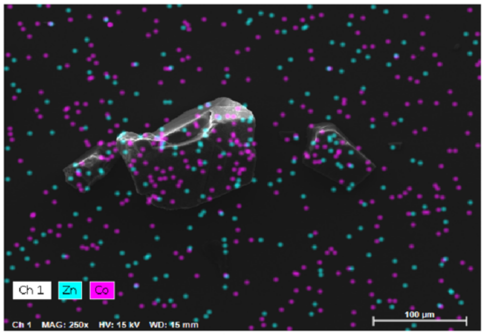

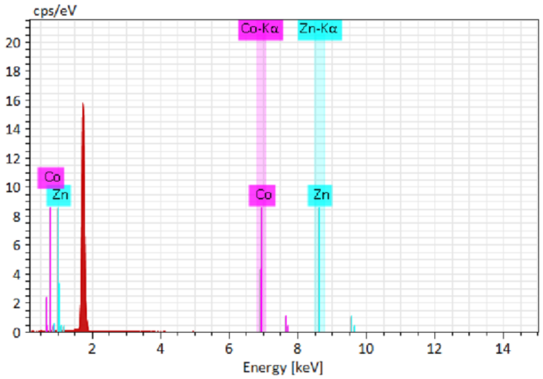

Element	Atomic Number	Weight [%]	Atomic [%]
Zn	30	17.31	24.11
Co	27	72.69	75.89
		100	100

Figure S21. SEM images and EDS-mapping data for mixed Zn(II)/Co(II) ALP-MOFs with varying Zn/Co ratios: 0.89:0.11 (a) , 0.58:0.42 (b), 0.76:0.24 (c). The numbers of each Zn/Co ratio result is the average value of three analyses.

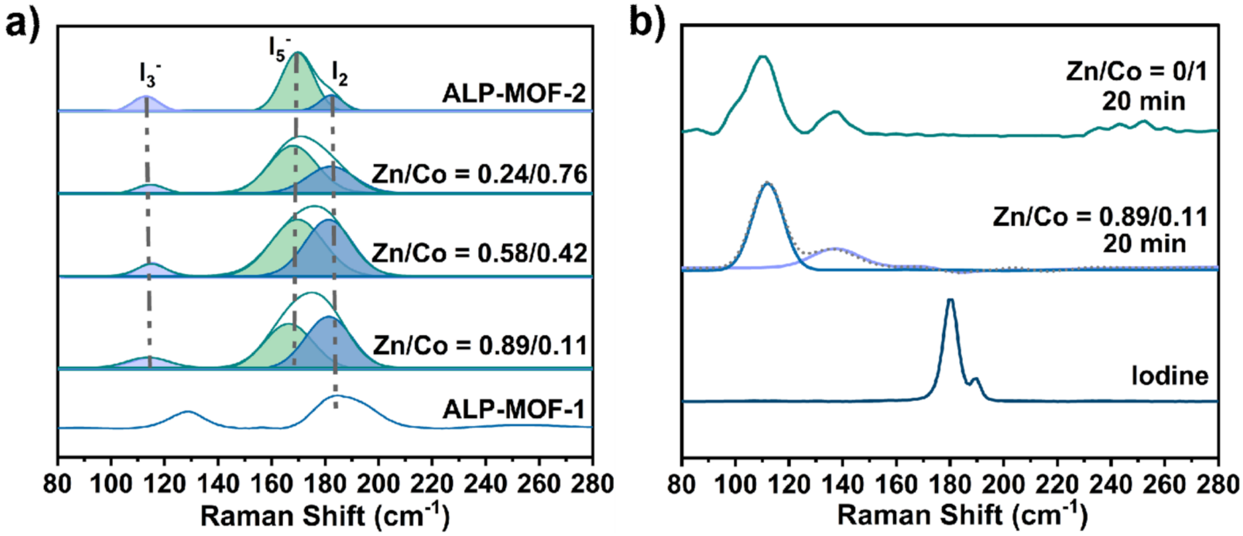

Figure S22. (a) Raman spectra for ALP-MOF-1, -2 and mixed Zn(II)/Co(II) ALP-MOFs with varying Zn/Co ratios after iodine adsorption saturation. (b) Raman spectra for pure iodine, the mixed Zn(II)/Co(II) ALP-MOF with the Zn/Co ratio of 0.89:0.11 and the Zn/Co = 0/1 analogue (ALP-MOF-2) after 20 min iodine adsorption.

Section 5. Crystallographic Data

5.1 Asymmetric Unit ORTEP Diagrams

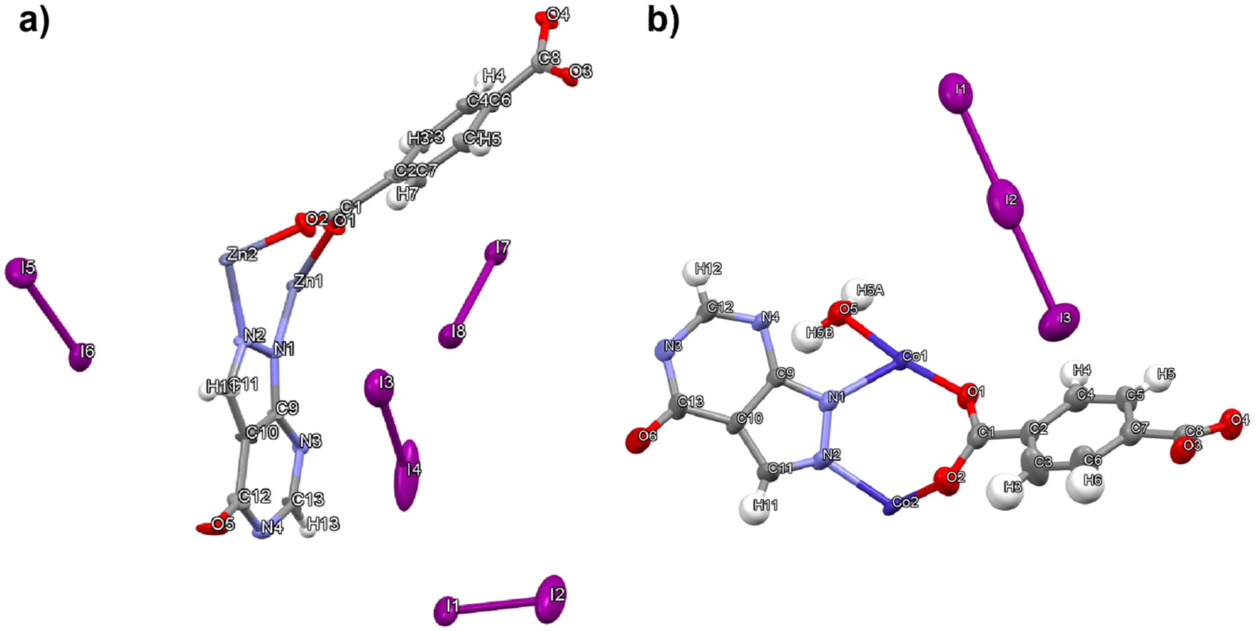

Figure S23. ORTEP plot of (a) ALP-MOF-1·2.97I_2 and (b) ALP-MOF-2·0.267I_3^- asymmetric unit. All non-hydrogen atoms are represented by thermal ellipsoids drawn at 50% probability level.

5.2 Crystallographic Tables

Table S4. Crystallographic data and structural refinement.

	ALP-MOF-1·2.97I_2	ALP-MOF-2·0.267I_3^-
Empirical formula	$C_{13}H_6I_{5.94}N_4O_5Zn_2$	$C_{13}H_8I_{0.8}N_4O_6Co_2$
Formula weight	1183.31	535.61
Crystal system	Hexagonal	Hexagonal
Space group	*R*-3	R-3
Unit cell dimensions (Å)	a = b = 35.4610(6), c = 12.7893(5)	a = b = 35.8099(16), c = 12.4261(10)
Volume (Å^3)	13927.7(7)	13799.8(17)
Z	18	18
Density (calculated)	2.539 Mg/m^3	1.160 Mg/m^3
Absorption coefficient	48.696 mm^{-1}	1.909 mm^{-1}
F(000)	9487	4651
Index ranges	-42≤h≤42, -41≤k≤42, -15≤l≤15	-39≤h≤43, -42≤k≤43, -14≤l≤14
Reflections collected	38080	36692
Independent reflections	5697 [R(int) = 0.1054]	5620 [R(int) = 0.2259]
Completeness	99.9%	99.9%
Absorption correction	Multi scan	Multi scan
Refinement method	Full-matrix least-squares on F^2	Full-matrix least-squares on F^2
Data / restraints / parameters	5697 / 0 / 295	5620 / 1 / 254
Goodness-of-fit on F^2	1.062	1.042
Final R indices [I>2sigma(I)]	R1 = 0.0995, wR2 = 0.2666	R1 = 0.0847, wR2 = 0.2581
R indices (all data)	R1 = 0.1173, wR2 = 0.2801	R1 = 0.1126, wR2 = 0.2753

	ALP-MOF-2·2H_2O
Empirical formula	$C_{13}H_{12}Co_2N_4O_8$
Formula weight	470.13
Crystal system	Hexagonal
Space group	*R*-3
Unit cell dimensions (Å)	a = b = 35.9018(11), c = 12.4515(6)
Volume ($Å^3$)	13899.0(9)
Z	18
Density (calculated)	1.011 Mg/m^3
Absorption coefficient	8.690 mm^{-1}
F(000)	4248
Index ranges	-44≤h≤44, -39≤k≤44, -15≤l≤15
Reflections collected	80152
Independent reflections	6064 [R(int) = 0.1220]
Completeness	99.5%
Absorption correction	Multi scan
Refinement method	Full-matrix least-squares on F^2
Data / restraints / parameters	6064 / 6 / 256
Goodness-of-fit on F^2	1.115
Final R indices [I>2sigma(I)]	R1 = 0.1165, wR2 = 0.1384
R indices (all data)	R1 = 0.3100, wR2 = 0.3322

$^aR_1 = \Sigma||F_o| - |F_c||/\Sigma|F_o|$. $^b wR_2 = [\Sigma w(F_o^2 - F_c^2)^2/\Sigma w(F_o^2)^2]^{1/2}$
$w = 1/[\sigma^2(F_o^2) + (aP)^2 + bP]$, $P = (F_o^2 + 2F_c^2)/3$.

Table S5. Selected bond lengths (Å) for iodic species in ALP-MOFs.

**ALP-MOF-1·2.97I_2**		**ALP-MOF-2·0.267I_3^-**	
I_1-I_2	2.664	I_1-I_2	2.809
I_3-I_4	2.686	I_2-I_3	3.015
I_5-I_6	2.693		
I_7-I_8	2.683		
